# Oxidative decomposition mechanisms of lithium carbonate on carbon substrates in lithium battery chemistries

**DOI:** 10.1038/s41467-022-32557-w

**Published:** 2022-08-20

**Authors:** Deqing Cao, Chuan Tan, Yuhui Chen

**Affiliations:** State Key Laboratory of Materials-Oriented Chemical Engineering, Nanjing Tech University, Nanjing, Jiangsu 211816 China

**Keywords:** Batteries, Batteries, Mass spectrometry

## Abstract

Lithium carbonate plays a critical role in both lithium-carbon dioxide and lithium-air batteries as the main discharge product and a product of side reactions, respectively. Understanding the decomposition of lithium carbonate during electrochemical oxidation (during battery charging) is key for improving both chemistries, but the decomposition mechanisms and the role of the carbon substrate remain under debate. Here, we use an in-situ differential electrochemical mass spectrometry-gas chromatography coupling system to quantify the gas evolution during the electrochemical oxidation of lithium carbonate on carbon substrates. Our results show that lithium carbonate decomposes to carbon dioxide and singlet oxygen mainly via an electrochemical process instead of via a chemical process in an electrolyte of lithium bis(trifluoromethanesulfonyl)imide in tetraglyme. Singlet oxygen attacks the carbon substrate and electrolyte to form both carbon dioxide and carbon monoxide—approximately 20% of the net gas evolved originates from these side reactions. Additionally, we show that cobalt(II,III) oxide, a typical oxygen evolution catalyst, stabilizes the precursor of singlet oxygen, thus inhibiting the formation of singlet oxygen and consequent side reactions.

## Abstract (web summary)

Lithium carbonate is ubiquitous in lithium battery chemistries and leads to overpotentials, however its oxidative decomposition is unclear. Here, the authors study its decomposition in ether electrolyte, clarify the role of the carbon substrate, and propose a route to limit released singlet oxygen.

## Introduction

Lithium carbonate (Li_2_CO_3_) is involved in many electrochemical systems, such as lithium-oxygen (Li-O_2_) batteries^1–15^, lithium-carbon dioxide (Li-CO_2_) batteries^16–30^, and lithium-ion (Li-ion) batteries^31–43^. Li_2_CO_3_ has extremely low ionic and electronic conductivity due to its wide bandgap^44,45^. In Li-O_2_ batteries, Li_2_CO_3_ mainly results from the side reactions of the reduced oxygen species attacking the electrolytes^1–3^. It not only passivates the electrode surface and polarizes the cell but also consumes the electrolyte, leading to electrolyte depletion and premature cell death. Therefore, Peng et al. referred to Li_2_CO_3_ as the “Achilles’ Heel” because it dominates the electrochemical performance of cells^3^. The accumulation of Li_2_CO_3_ during cycling has to be well-addressed and resolved in the pursuit of high-performance Li-O_2_ batteries. In Li-CO_2_ batteries, Li_2_CO_3_ is the main desirable discharge product, but the Li_2_CO_3_ decomposition during charging has sluggish kinetics and requires a large overpotential. Many efforts have been devoted to designing highly efficient catalysts to reduce the large overpotential^18–29^. In both Li-O_2_ batteries and Li-CO_2_ batteries, Li_2_CO_3_ in the composite electrode needs to be oxidatively decomposed during the charging process, otherwise, it passivates the electrode surface and kills the cells. However, the mechanism of Li_2_CO_3_ decomposition is still unclear and this would seriously hinder the research progress. For instance, the role of carbon in the charging process is still under debate. In Li-ion batteries, Li_2_CO_3_ is one of the main components of the solid electrolyte interphase of the anode and exists as a surface contaminant present on lithium transition metal oxides used in the cathode thus it influences the cell performance^32^. For instance, the lithium transition metal oxides cathode materials are usually covered with a layer of Li_2_CO_3_ due to the residual lithium precursors reacting with CO_2_ from the ambient atmosphere^33–35^. Very recently, McCloskey and co-workers have studied the Li_2_CO_3_ decomposition mechanism on Li-ion cathodes. Using isotopic labeling, they found that when Li_2_CO_3_ is present at the cathode surface, organic fragments containing diatomic oxygen are formed on the cathode surface during the charging process above 4.2 V versus Li^+^/Li and the diatomic oxygen within these fragments mainly originates from the lithium transition metal oxides lattice and only a minor fraction originates from the Li_2_CO_3_ itself^33–35^. In summary, the decomposition of Li_2_CO_3_ is so important that it determines the electrochemical performance in many systems. However, its mechanisms are still controversial and not yet well understood, even in the Li-CO_2_ cells. Li_2_CO_3_ decomposition during the charging process is generally divided into two types, chemical routes, and electrochemical routes^41,42^. Recently, Freiberg et al claimed that Li_2_CO_3_ decomposition in lithium hexafluorophosphate (LiPF_6_)-ethylene carbonate (EC)-ethylmethyl carbonate (EMC) electrolyte follows a chemical route reacting with H^+^, which is induced by electrolyte oxidation at >4.6 V^41^. In contrast, Mahne et al. claimed that Li_2_CO_3_ decomposition is an electrochemical process^33,46^. Here, our results show that the electrolyte salt affects the route and the chemical reaction is likely caused by LiPF_6_, which will be discussed later.

So far, several electrochemical mechanisms have been proposed and four possible reaction pathways are summarized below:

1 $$\begin{array}{cc}{{{{{{\rm{Li}}}}}}}_{2}{{{{{{\rm{CO}}}}}}}_{3}-2{{{{{{\rm{e}}}}}}}^{-}\,\to \,2{{{{{{\rm{Li}}}}}}}^{+}+{{{{{{\rm{CO}}}}}}}_{2}+1/2\,{{{{{{\rm{O}}}}}}}_{2} & {{{{{{\rm{E}}}}}}}_{{{{{{\rm{rev}}}}}}}=3.8\,{{{{{\rm{V}}}}}}\end{array}$$

1b $$\begin{array}{cc}{{{{{{\rm{Li}}}}}}}_{2}{{{{{{\rm{CO}}}}}}}_{3}-2{{{{{{\rm{e}}}}}}}^{-}\to 2{{{{{{\rm{Li}}}}}}}^{+}+{{{{{{\rm{CO}}}}}}}_{2}+1/2{}^{1}{{{{{\rm{O}}}}}}_{2} & \,{{{{{{\rm{E}}}}}}}_{{{{{{\rm{rev}}}}}}}=4.1\,{{{{{\rm{V}}}}}}\end{array}$$

2 $$\begin{array}{cc}2{{{{{{\rm{Li}}}}}}}_{2}{{{{{{\rm{CO}}}}}}}_{3}+{{{{{\rm{C}}}}}}-4{{{{{{\rm{e}}}}}}}^{-}\to 4{{{{{{\rm{Li}}}}}}}^{+}+3{{{{{{\rm{CO}}}}}}}_{2} & {{{{{{\rm{E}}}}}}}_{{{{{{\rm{rev}}}}}}}=2.8\,{{{{{\rm{V}}}}}}\end{array}$$

3 $$2{{{{{{\rm{Li}}}}}}}_{2}{{{{{{\rm{CO}}}}}}}_{3}-3{{{{{{\rm{e}}}}}}}^{-}\,\to \,4{{{{{{\rm{Li}}}}}}}^{+}+2{{{{{{\rm{CO}}}}}}}_{2}+{{{{{{{\rm{O}}}}}}}_{2}}^{\cdot -}$$

In Eq. (1a and b), both CO_2_ and O_2_ formed and it takes 2e^–^ per CO_2_ molecule^16,17^. The only difference is that O_2_ forms as a triplet O_2_ in Eq. (1a) and as a singlet O_2_ (^1^O_2_) in Eq. (1b), respectively. ^1^O_2_ has been detected at a charging voltage above 3.8 V in the oxidation of Li_2_CO_3_, by using high-performance liquid chromatography (HPLC) and nuclear magnetic resonance spectrometry (^1^H NMR) analysis^46^. In Eq. (2), carbon and Li_2_CO_3_ were oxidized together to form CO_2_ via a 4e^–^ process. That is a common mechanism proposed for the charging process of Li-CO_2_ cells. In fact, Eq. (2) is unlikely to be an elemental reaction, which will be discussed in the text later. In Eq. (3), oxygen is released in the form of the superoxide radical. Qiao et al.^27^ used in situ surface-enhanced Raman spectroscopy to observe the dimethyl sulfone during the charging process and they explained that dimethyl sulfone is attributed to the nucleophilic attack on DMSO solvent from reduced oxygen species (superoxide radicals etc.).

Despite the above progress, researchers mainly focus on Li_2_CO_3_ and little attention has been paid to the carbon in the charging process. Carbon is always added to the composite electrodes as the conductive additives, however, it is always neglected and its role in Li_2_CO_3_ decomposition has not been considered yet. Overall, the mechanism of Li_2_CO_3_ decomposition is still under debate and the role of carbon is mysterious.

Here, we labeled the Li_2_CO_3_ and carbon substrate with ^13^C-isotope and qualitatively analyzed the gas products from the decomposition of Li_2_CO_3_, carbon, and electrolyte, respectively. We quantified the gas evolution, particularly CO, during the charging process using an in situ differential electrochemical mass spectroscopy-gas chromatography (DEMS-GC) coupling system. We found that Li_2_CO_3_ decomposition is mainly an electrochemical process rather than a chemical process induced by electrolyte oxidation. Li_2_CO_3_ decomposes to CO_2_ only, but no CO nor O_2_. The oxygen from Li_2_CO_3_ is released as highly reactive ^1^O_2_, which further attacks the electrolyte and carbon substrate in the composite electrodes to form CO_2_ and CO.

## Results and discussion

To study the oxidative decomposition process of Li_2_CO_3_, a cell with a Li_2_CO_3_-carbon composite electrode was constructed and charged. Li_2_CO_3_ was electro-oxidized and the gas evolution was quantified. Because both CO and N_2_ have the same mass-to-charge ratio of 28 (*m/z* = 28), the mass spectrometer typically used in a DEMS system lacks sufficient mass resolution to distinguish the contribution from CO (*m/z* = 28.0104) and N_2_ (*m/z* = 28.0140). Although GC could separate the CO and N_2_, it cannot distinguish and quantify the ^13^CO_2_/^13^CO_2_ and ^12^CO/^13^CO. Therefore, an in situ DEMS-GC coupling system (Supplementary Fig. 1) was used to quantify the evolution of ^12^CO, ^12^CO_2_, ^13^CO, and ^13^CO_2_. The details of the experiments are described in Methods. As shown in Supplementary Fig. 2, after calibration, the CO evolution signals from DEMS and GC experiments are consistent, which provides a reliable amount of CO in the following experiments.

**Fig. 1:**
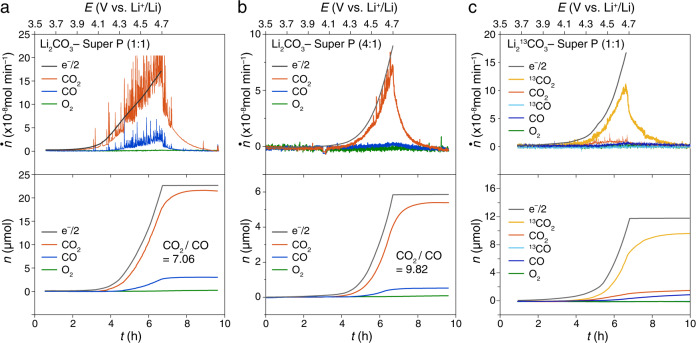
Gas evolution of oxidative decomposition of Li_2_CO_3_ and Li_2_^13^CO_3_. The gas evolution during the charging process of the cells using **a** Li_2_CO_3_-Super P (1:1), **b** Li_2_CO_3_-Super P (4:1), **c** Li_2_^13^CO_3_-Super P (1:1) composite electrodes in 1 M LiTFSI-tetraglyme. Ar flow rate: 0.5 mL min^−1^. Sweep rate: 0.05 mV s^−1^. The molar flux (top panel) of gas evolution was denoted as *ṅ* and the charging current is translated to *ṅ*_electron_ and the cumulative mole (bottom panel) of the gas was denoted as *n*.

Super P carbon (Timcal) was ball milled with Li_2_CO_3_ with a mass ratio of 1:1. A Li_2_CO_3_-Super P (1:1) composite electrode was prepared to construct a cell with 1 M lithium bis(trifluoromethane-sulphonyl)imide (LiTFSI)—tetraglyme electrolyte as stated in Methods and the cells were charged by linear sweep voltammetry (LSV). As shown in Fig. 1a, the anodic current for oxidation reaction and gas evolution started at 3.9 V (vs. Li^+^/Li, all potentials in the text below are versus Li^+^/Li), which is consistent with the thermodynamic decomposition potential of Li_2_CO_3_ (3.82 V according to Eq. 1). A control experiment without Li_2_CO_3_ was carried out (Supplementary Fig. 3). The onset potential of electrolyte decomposition is at 4.3 V. The background current of carbon/electrolyte oxidation at 3.8 V is 10-fold smaller than Li_2_CO_3_ decomposition (Fig. 1a) and there is no CO_2_ or O_2_ evolution. In Fig. 1a, a large amount of CO_2_ and CO were identified as the gas products, which confirms the decomposition of Li_2_CO_3_, consistent with the literature^16,17^. According to the mass loading of the electrode (see Methods), ~60% of the preloaded Li_2_CO_3_ decomposed eventually. The ratio e-/CO_2_ for Fig. 1a is 2.1, close to 2e^–^ per CO_2_ (Supplementary Table 1), suggesting it is an electrochemical process. The deviation is from the electrolyte electro-oxidation without producing CO_2_ (Supplementary Fig. 3), which is consistent with the literature^41^. The molar flux of both CO_2_ (denoted as *ṅ*_CO2_) and CO (denoted as *ṅ*_CO_) follow the trend of the current during charging (equivalent to *ṅ*_electron_) but they could derive from different processes because of the multisource of the CO_2_ and CO evolution. Therefore, we mainly focus on the ratio CO_2_/CO because it helps us to determine the mechanisms (Eqs. 1–3). The ratio CO_2_/CO exhibits the comparison between Li_2_CO_3_ decomposition and other reactions because Li_2_CO_3_ decomposition does not form CO, as discussed later. The ratio CO_2_/CO in Fig. 1a is 7.06 (Supplementary Table 2), which does not fit any reaction pathways proposed above. Due to the lack of O_2_ evolution, Eq. (1a) could be excluded. The oxygen appears as singlet O_2_ (^1^O_2_) instead of low-energy triplet O_2_ (^3^O_2_), which will be discussed in detail later.

To further study the influence of electrode composition on the gas products, the same experiment was conducted using a Li_2_CO_3_-Super P (4:1) composite electrode. As shown in Fig. 1b, similar gas species without O_2_ were identified. Because the Li_2_CO_3_-Super P (4:1) composite electrode contains more insulating Li_2_CO_3_ solid than that in Li_2_CO_3_-Super P (1:1) electrode, leading to poor solid-solid contact, the charging current and consequent gas evolution in Li_2_CO_3_-Super P (4:1) is one order of magnitude lower than those in Li_2_CO_3_-Super P (1:1) electrode. If the CO_2_ and CO came from a certain intermediate or a simple one-step reaction, the same ratio CO_2_/CO would be observed. However, the ratio CO_2_/CO varies with the electrode composition, from 7.06 to 9.82 (Supplementary Table 2). This result suggests that the Li_2_CO_3_ decomposition is a complicated multistep process, instead of a simple one-step reaction as proposed previously^16,17^. Because Li_2_CO_3_, the carbon substrate, and the electrolyte all might contribute to CO_2_ and CO evolution, it brings up the confusion about the source of CO_2_ and CO, which is the key to addressing the reaction mechanisms.

### Gas products of Li_2_^13^CO_3_ decomposition

The isotope-labeled Li_2_^13^CO_3_ was used to identify the decomposition mechanisms of Li_2_CO_3_. The Raman spectrum and XRD pattern of the Li_2_^13^CO_3_ confirm its composition (Supplementary Fig. 4). The ^12^C-impurity in Li_2_^13^CO_3_ is determined by using a mass spectrometer (MS). The details are described in Methods—Isotope impurities. The Li_2_^13^CO_3_ contains 15% isotope impurity of Li_2_^12^CO_3_ (Supplementary Fig. 5), which would be subtracted from the MS results in the following experiments.

Figure 1c exhibits the gas evolution during the charging process of the cell with a Li_2_^13^CO_3_-Super P composite electrode. Li_2_^13^CO_3_ was electro-oxidized and a large amount of ^13^CO_2_ is identified, which is definitely from the Li_2_^13^CO_3_ decomposition because both Super P carbon and electrolyte are unlabeled. No O_2_ evolution is identified again. ^13^CO was not observed as well, indicating that the Li_2_^13^CO_3_ decomposed to merely ^13^CO_2_ without ^13^CO. The decomposition of Li_2_^13^CO_3_ (releasing ^13^CO_2_) contributes 79% of the overall CO_2_ and CO evolution and it is the dominant process during charging. Meanwhile, some CO_2_ and CO from the inevitable decomposition of electrolyte and carbon substrate were identified during charging. The electrolyte/carbon decomposition contributes to approximately one-fifth of total gas evolution (Supplementary Table 3), which is a high ratio of side reactions.

In the Li-CO_2_ chemistry with lithium carbonate isotopically labeled with ^13^C on an unlabeled carbon substrate (which we write here as ^12^C for simplicity), Li_2_^13^CO_3_ is posited to decompose together with the carbon substrate in the following reaction:

4 $$2{{{{{{\rm{Li}}}}}}}_{2}{\,\!}^{13}{{{{{\rm{C}}}}}}{{{{{{\rm{O}}}}}}}_{3}+{\,\!}^{12}{{{{{\rm{C}}}}}}-4{{{{{{\rm{e}}}}}}}^{-}\to 4{{{{{{\rm{Li}}}}}}}^{+}+{2}{\,\!}^{13}{{{{{{\rm{CO}}}}}}}_{2}+{\,\!}^{12}{{{{{\rm{C}}}}}}{{{{{{\rm{O}}}}}}}_{2}$$

If it was the case, the ratio between ^13^CO_2_ and ^12^CO_2_ is expected to be about 2/1, but we find a ratio of 6.1/1 (Supplementary Table 4), much higher than 2/1 because ^12^C does not transform to sufficient ^12^CO_2_. This result suggests that the decomposition of the Li_2_CO_3_-C electrode is a complicated reaction instead of a simple reaction with known stoichiometric numbers like Eq. (4). Therefore, the contribution of the C substrate during the charging process is the key to demystifying the reaction mechanisms.

### Decomposition of ^13^C-carbon substrate

**Fig. 2:**
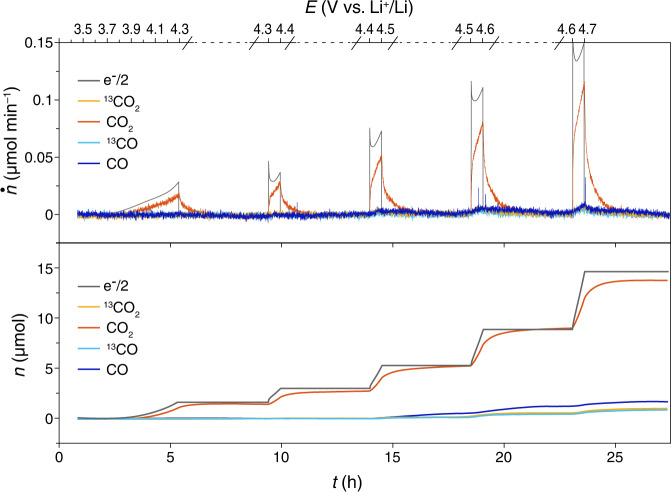
Potential-dependent CO_2_ and CO evolution during the charging process. The gas evolution during the charging process of the cells using Li_2_CO_3_-^13^C (1:1) composite electrode in 1 M LiTFSI-tetraglyme. Sweep rate: 0.05 mV s^−1^. The cell rested for 4 h after reaching 4.3, 4.4, 4.5, 4.6, and 4.7 V, respectively. Ar flow rate: 0.5 mL min^−1^. The molar flux (top panel) of gas evolution was denoted as *ṅ* and the charging current is translated to *ṅ*_electron_ and the cumulative mole (bottom panel) of the gas was denoted as *n*.

To determine the contribution of carbon in this reaction, the same experiments were carried out again but by replacing the Super P carbon substrate with ^13^C-carbon and leaving the lithium carbonate and electrolyte unlabeled. The ^13^C-carbon contains 1.5% of ^12^C impurity as shown in Supplementary Fig. 6. As shown in Fig. 2 and Supplementary Fig. 7, both ^13^CO_2_ and ^13^CO evolved during the charging process and the rest of CO_2_ and CO evolution came from the decomposition of Li_2_CO_3_ and electrolyte. As the only source of ^13^C-isotope, ^13^C is oxidized to release both ^13^CO_2_ and ^13^CO during the charging process. The formation of ^13^CO exhibits that the ^13^C is oxidized incompletely like an incomplete combustion reaction. The ^13^C substrate could be oxidized either electrochemically by potential or chemically by oxidative agents that are formed in the previous steps. If ^13^C is oxidized electrochemically, the ratio ^13^CO_2_/^13^CO would depend on the potential (Fig. 2). If ^13^C is oxidized chemically by oxidative agents like ^1^O_2_ and superoxide species, which are generated during the charging process, the electrode composition affects the ratio between ^13^CO_2_ and ^13^CO.

### Impact of the potential on the decomposition of ^13^C-carbon substrate

Due to the high equilibrium potential of Li_2_CO_3_ decomposition (3.82 V), the cell potential could affect the decomposition reactions and thus affect the formation of ^13^CO_2_ and ^13^CO at different potentials during the charging process. Therefore, a cell with Li_2_CO_3_- Super P composite electrode was charged with LSV from 4.3 to 4.7 V and the gas evolution was measured. The cell was rested for 4 h after reaching 4.3, 4.4, 4.5, 4.6, and 4.7 V, respectively, to obtain a low background of gas evolution. The cumulative molar of gas evolution at various potential stages is shown in Fig. 2. The amount of CO_2_ from Li_2_CO_3_ decomposition increased when the potential increased. The higher the potential was, the more CO_2_ evolved, in accord with the accelerating decomposition of Li_2_CO_3_. Meanwhile, ^13^CO_2_ and ^13^CO evolved simultaneously and their amount increased together with the CO_2_ evolution when the potential increased. Supplementary Fig. 7 shows the gas evolution at various stages of the charging process from 4.4 to 4.7 V. The amount of ^13^CO_2_ and ^13^CO evolution increased with the rising potential up to 4.7 V. This increase is probably because more singlet oxygen forms at a higher potential, which leads to more severe carbon and electrolyte degradation. Although both ^13^CO_2_ and ^13^CO increased, the ratio ^13^CO_2_/^13^CO remains almost identical at these stages. This result suggests that the ^13^CO_2_ and ^13^CO likely originated from the same chemical reaction.

To check whether ^13^C is oxidized to any solid byproducts, the cell was dissembled at the end of charging and the composite electrode was collected to further quantify the remained solid by-product of inorganic/organic carbon as described in Methods—Quantification of the solid byproducts. Neither Li_2_^13^CO_3_ (Supplementary Fig. 8a) nor organic ^13^C-carbonates (Supplementary Fig. 8b) were detected in the charged electrode, indicating that the ^13^C did not oxidize to any solid by-products, i.e., inorganic and organic carbonates. Only unlabeled inorganic Li_2_CO_3_ and organic carbonate were identified, which are from undecomposed Li_2_CO_3_ and electrolyte decomposition. The incomplete decomposition of Li_2_CO_3_ may be due to the poor solid-solid contact between the carbon substrate and Li_2_CO_3_ during the charging process. It is unlikely to decompose all Li_2_CO_3_ even up to 4.7 V, which is consistent with the literature^41^. Therefore, as only ^13^CO and ^13^CO_2_ and no ^13^C-containing solid products are detected when using ^13^C as the carbon substrate, the carbon substrate is only oxidized to gaseous carbon monoxide and carbon dioxide during charge in the presence of lithium carbonate and the 1 M LiTFSI-tetraglyme electrolyte.

Previous results showed that the functional groups at the carbon surface dominated the decomposition of the carbon substrate^47^. Therefore, the Raman and XPS spectra of ^13^C and Super P are recorded to show their surface condition (Supplementary Fig. 9). D-band and G-band of ^13^C were observed at 1289 and 1507 cm^–1^, respectively, in the Raman spectrum (Supplementary Fig. 9a). Both D-band and G-band drift in the negative direction due to the isotope effect. The intensity of the D-band and G-band (*I*_*D*_*/I*_*G*_) typically represents the disorderliness and amounts of defects in the carbon structure. Here, ^13^C and Super P carbon show the same *I*_*D*_*/I*_*G*_ of 1.20, which indicates the same degree of disorderliness and a similar amount of defects from the surface of ^13^C and Super P. Similar XPS spectra are shown in Supplementary Fig. 9b, confirming the similar surface groups of these two carbon substrates. Therefore, ^3^C and Super P have similar surface functional groups and they are likely to exhibit similar behavior in this carbon decomposition on charge.

### Impact of the ratio of Li_2_CO_3_ and carbon on the gas evolution

**Fig. 3:**
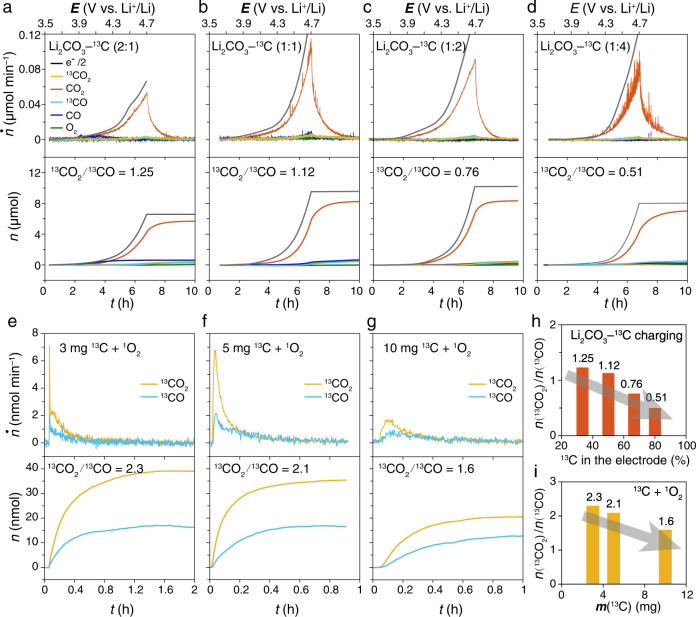
Incomplete oxidation of ^13^C-carbon by singlet O_2_ to form ^13^CO and ^13^CO_2_. **a**–**d** The gas evolution during the charging process of the cells using the Li_2_CO_3_-^13^C composite electrodes with various mass ratios of Li_2_CO_3_/^13^C, **a** 2:1, **b** 1:1, **c** 1:2, **d** 1:4 in 1 M LiTFSI-tetraglyme. **e**–**g** ^13^CO_2_ and ^13^CO evolution of the ex situ chemical reaction between singlet O_2_ and ^13^C-carbon in tetraglyme solution. 22 mg KO_2_ powder was mixed with **e** 3 mg, **f** 5 mg, **g** 10 mg of ^13^C-carbon, respectively, and then 1 mL of LiTFSI-tetraglyme(4 M) was added to react with KO_2_ to form singlet O_2_. The comparison of ratio ^13^CO_2_/^13^CO in (**h**) ex situ chemical reaction for (**e**–**g**) and **i** charging process for (**a**–**d**).

Although the ^13^C carbon substrate oxidized to ^13^CO and ^13^CO_2_, its decomposition pathway is still ambiguous. It might be a pathway similar to the incomplete combustion reaction. Alternatively, ^13^CO and ^13^CO_2_ might be derived from a reaction intermediate with known structures like oxalate (C_2_O_4_^2−^). To clarify the pathway, we studied the gas products of four Li_2_CO_3_-^13^C composite electrodes with various mass ratios of Li_2_CO_3_/^13^C from 2:1 to 1:4 (Fig. 3). Overall, the carbon decomposition contributes to about 10% of the total gas evolution and the exact contribution depends on the composition of the electrodes (Supplementary Table 3). When the composite electrode contains less Li_2_CO_3_, ratio ^13^CO_2_/^13^CO decreases from 1.25 to 0.51 (Fig. 3h), indicating the extent of oxidation of ^13^C is restricted by the amount of Li_2_CO_3_ in the composite electrodes. Otherwise, the ^13^C would be completely oxidized to ^13^CO_2_ instead of the mixture of ^13^CO_2_ and ^13^CO. This varying ratio ^13^CO_2_/^13^CO confirms that the overall reaction is a multistep reaction, rather than forming a complex intermediate like C_2_O_4_^2−^ which should give a certain ratio ^13^CO_2_/^13^CO independent of the ratio of Li_2_CO_3_/^13^C. The ^13^C is highly likely to be oxidized by the oxidative intermediates from Li_2_CO_3_ decomposition. Therefore, as ratio ^13^CO_2_/^13^CO varied with varying composition of the Li_2_CO_3_-^13^C composite electrode, we argue that the amount of Li_2_CO_3_ present in the electrode, and thus the amount of oxidative intermediates from Li_2_CO_3_ decomposition, influences the decomposition route of the carbon substrate.

### Oxidation of ^13^C-carbon by ^1^O_2_ and superoxide

Both ^1^O_2_ and superoxide are reactive intermediates formed during the discharge and charging process in Li-air batteries, which could result in the decomposition of the carbon substrate^48–52^. ^1^O_2_ could attack the electrolytes and the electrodes as a strong oxidative species. Here, 9,10-dimethylanthracene (DMA), a molecular trap for ^1^O_2_, is used to identify the ^1^O_2_ during electrochemical oxidation for the Li_2_CO_3_-C composite electrodes (see Methods—Identification of ^1^O_2_). When ^1^O_2_ forms, it rapidly reacts with DMA to form DMAO_2_, which could be identified in the ^1^H NMR spectrum. Here, at the end of the charging process, the electrolyte with DMA in the cell was extracted and its ^1^H NMR spectrum (Supplementary Fig. 10) shows that DMAO_2_ has formed, which confirms the formation of ^1^O_2_ during the charging process. Once ^1^O_2_ forms, ^1^O_2_ attacks carbon and releases ^13^CO and ^13^CO_2_.

Here, to study the contribution of ^1^O_2_ and KO_2_ to carbon decomposition, ^1^O_2_ and KO_2_ were respectively used to react with ^13^C as ex situ chemical experiments. ^1^O_2_ is produced from the disproportionation of superoxide species^47^ (The reaction between KO_2_ and Li^+^ in this work). More details are described in Methods—Chemical experiments between reactive oxygen species and carbon. The gas evolutions of ^13^CO_2_, ^13^CO, ^12^CO_2_, and O_2_ in the reaction between ^1^O_2_ and ^13^C are shown in Fig. 3e–g and Supplementary Fig. 11. Figure 3e–g show both ^13^CO and ^13^CO_2_ evolution, which confirms the incomplete oxidation of ^13^C-carbon by ^1^O_2_. The formation efficiency of ^1^O_2_ by disproportionation in solution is low, therefore, most O_2_ is released as ^3^O_2_ (Supplementary Fig. 11). Because ^1^O_2_ is formed in the electrolyte solution rather than at the surface of ^13^C, fresh ^1^O_2_ is more likely to attack the electrolyte than the ^13^C, forming a large amount of CO_2_. In addition, because a large amount of carbon makes the suspension viscous, the total amount of ^13^CO and ^13^CO_2_ slightly decreased with the increase of ^13^C. When the amount of ^13^C increased, more ^13^CO formed and the ratio ^13^CO_2_/^13^CO decreased (Fig. 3i and Supplementary Table 5). Both the in situ electrochemical charging experiments (Fig. 3h) and ex situ chemical experiments (Fig. 3i) show the same decreasing trend of ratio ^13^CO_2_/^13^CO with the increase of the amount of ^13^C relative to ^1^O_2_ or Li_2_CO_3_.

On the other hand, to further exclude the contribution from superoxide species, the same chemical experiments were carried out but replaced ^1^O_2_ with O_2_^–^_(sol)_. KO_2_ was dissolved in tetraglyme and crown ether was added to maximize the concentration of the O_2_^−^_(sol)_ in the solution. As shown in Supplementary Fig. 12, only ^13^CO but no ^13^CO_2_ was identified, which suggests that O_2_^−^_(sol)_ is incapable to oxidize ^13^C to ^13^CO_2_. In summary, the detected ^13^CO and ^13^CO_2_ come from the side-reaction of ^1^O_2_ attacking the ^13^C substrate (Eq. 6). Herein, we simulate the chemical reaction between ^1^O_2_ and ^13^C without applying potential, however, the potential applied in the charging process could make the real side-reaction more complicated. More ^1^O_2_ might be produced at a higher potential. It is noted that oxygenated byproducts from chemical reactions with ^1^O_2_ could first be produced and then electro-oxidatively decomposed^48,49^.

### The CO and CO_2_ evolution of electrolyte decomposition

**Fig. 4:**
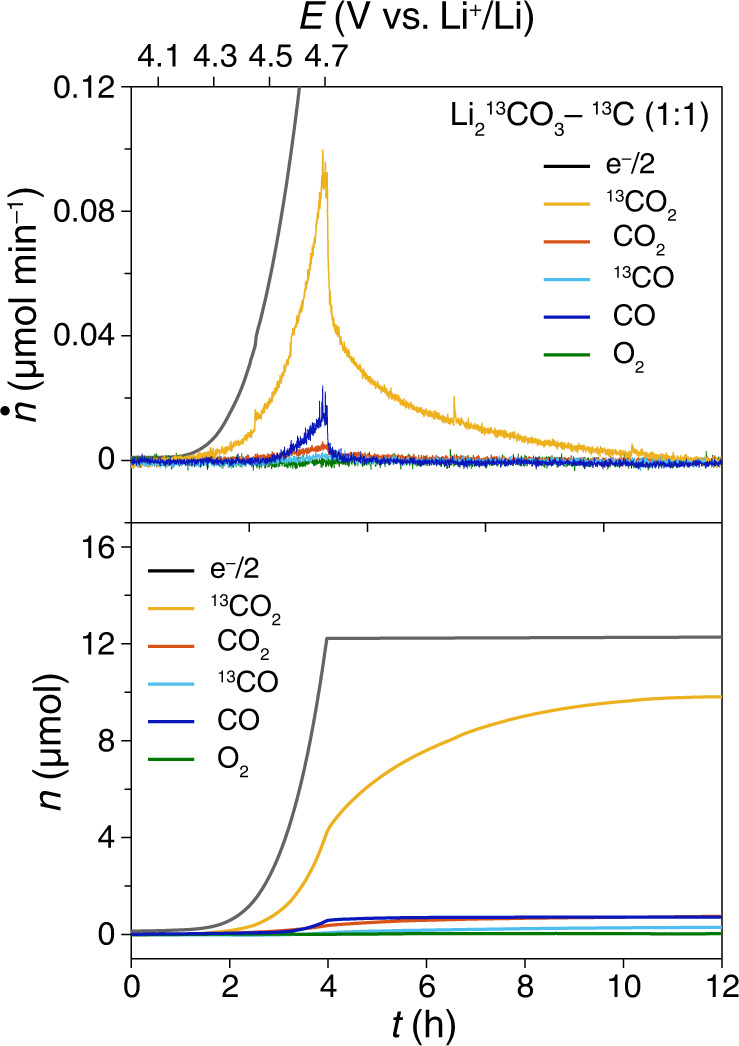
The gas evolution of the electrolyte decomposition. The gas evolution during the charging process of the cells using Li_2_^13^CO_3_-^13^C-carbon (1:1) composite electrode in 1 M LiTFSI-tetraglyme. Ar flow rate: 0.5 mL min^−1^. Sweep rate: 0.05 mV s^−1^. The molar flux (top panel) of gas evolution was denoted as *ṅ* and the charging current is translated to *ṅ*_electron_ and the cumulative mole (bottom panel) of the gas was denoted as *n*. The unlabeled CO_2_ and CO are contributed by electrolyte decomposition.

The electrolyte decomposition during the oxidation of Li_2_CO_3_ on charge is inevitable and should not be ignored. A cell with Li_2_^13^CO_3_-^13^C composite electrode was charged to quantify the contribution from electrolyte degradation (Fig. 4). Because the entire composite electrode is labeled with ^13^C isotope, the ^12^CO_2_ must come from the decomposition of the tetraglyme electrolyte. Figure 4 shows that ^12^CO_2_ and ^12^CO from the electrolyte decomposition contribute ~12% to the total gas evolution (Supplementary Table 6). The ratio between carbon dioxide species (^12^CO_2_ + ^13^CO_2_) and carbon monoxide species (^12^CO + ^13^CO) is 7.76, which is in accord with the ratio CO_2_/CO of 7.06 in the Li_2_CO_3_- Super P (1:1) composite electrode, as stated in Supplementary Table 2.

As discussed above, the decomposition of Li_2_CO_3_ and carbon substrate contributes to 79% and ~10%, respectively, of the total gas evolution. The side-reactions of carbon depend on the electrode composition because this charging process highly depends on the solid-solid contact between Li_2_CO_3_ and carbon substrate. The exact ratios of decomposition of individual components were not completely identical in each experiment because the nature of Li_2_CO_3_ decomposition is based on the solid-solid contact. At some contacting points, the charging process will stop due to the large overpotential caused by the restricted solid-solid contact. The sum of gas evolution from Li_2_CO_3_, carbon, and electrolyte is ~100%, which confirms the reliability of individual measurements.

Although an important consideration, full understanding of electrolyte decomposition is not our focus in this work. Therefore, to ensure that it does not affect the major gas evolution from Li_2_CO_3_ and C, we quantified the gas by-products and solid by-products from the electrolyte decomposition without further analyzing the liquid by-products dissolved in the solvent. The solid by-products, like Li_2_CO_3_ and organic carbon residuals in the composite electrode at the end of the charge, were quantified respectively. As shown in Supplementary Fig. 13, approximately one-third of Li_2_^13^CO_3_ is unreacted and remained in the electrode and a trace amount (<0.1 μmol) of organic carbonate were identified. This result indicates that there is no exchange of ^12^C/^13^C between the electrolyte and Li_2_CO_3_/C.

### Electrochemical vs. chemical pathways of Li_2_CO_3_ decomposition

Here our results show that Li_2_CO_3_ decomposition in the ether-based electrolyte is mainly an electrochemical process, which is in good agreement with Kaufman et al.^33^, but Freiberg et al claimed that Li_2_CO_3_ decomposition was a chemical process in LiPF_6_ carbonate-based electrolyte and it was induced by water^41^. They argued that the electrolyte was oxidized and decomposed on high potential and it formed some proton-derivatives which chemically react with Li_2_CO_3_ to evolve CO_2_. To study the reasons of these contradicting results, we conducted a series of comparative experiments. Firstly, to avoid the direct participation of water in Li_2_CO_3_ decomposition, we carried out a chemical reaction between the commercial Li_2_CO_3_ and 1 M LiTFSI tetraglyme electrolyte with 1000 ppm and 5000 ppm H_2_O, respectively, and the gas evolution was recorded. The result in Supplementary Fig. 14 indicates that no CO_2_ and O_2_ were detected even if there were lots of protons (from water) in the electrolyte. Then, to check the impact of potential, a cell with blank carbon (Super P or ^13^C) electrode without Li_2_CO_3_ but with 1000 ppm of H_2_O in the electrolyte and the gas evolution was recorded, Supplementary Fig. 15. No O_2_ and CO_2_ evolved during the charging process below 4.7 V. Interestingly, in the presence of 1000 ppm H_2_O, the onset potentials of electrolyte decomposition drifted to a lower potential of 3.8 V, compared with 4.1 V without H_2_O. This result implies that H_2_O encourages the electro-oxidation of the electrolyte but there is still no gas evolution below 4.5 V.

Learning from Freiberg’s work, we decomposed Li_2_CO_3_ without direct electric contact to confirm the chemical mechanism^41^. Briefly, an extra Celgrad membrane was placed between Li_2_CO_3_ powder and the blank carbon composite electrode to separate them. On one hand, the Li_2_CO_3_ cannot be electro-oxidized due to the lack of direct electronic contact with the carbon electrode. On the other hand, the porous Celgard membrane allows the electrolyte decomposition byproducts that form at the carbon composite electrode to diffuse across the Celgard and react with Li_2_CO_3_.

**Fig. 5:**
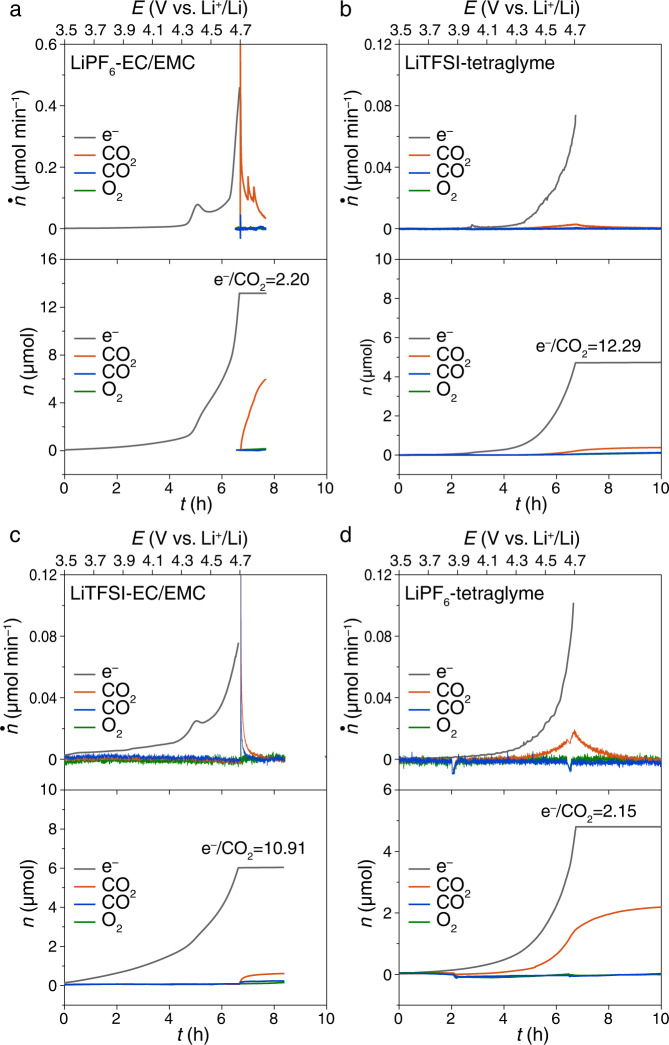
Impact of electrolyte salts and solvents on the gas evolution of chemical decomposition of Li_2_CO_3_. The gas evolution during the charging process of the cells using Super P-PTFE composite electrodes without direct contact of Li_2_CO_3_ in **a** 1 M LiPF_6_-EC/EMC, **b** 1 M LiTFSI-tetraglyme. **c** 1 M LiTFSI-EC/EMC, and **d** 1 M LiPF_6_-tetraglyme. 20 mg of commercial Li_2_CO_3_ was separated from the Super P-PTFE electrode with a Celgard separator to allow a chemical decomposition but not electro-oxidation decomposition. Ar flow rate: 0.5 mL min^−1^. Sweep rate: 0.05 mV s^−1^. The molar flux (top panel) of gas evolution was denoted as *ṅ* and the charging current is translated to *ṅ*_electron_ and the cumulative mole (bottom panel) of the gas was denoted as *n*.

For comparison, in the LiPF_6_-ethylene carbonate (EC)/ethyl methyl carbonate (DMC) electrolyte, the same setup was used but the gas evolution was analyzed at the end of the charging process due to the volatility of the EMC. Because the Li_2_CO_3_ is separated from the carbon electrode, it cannot be electro-oxidized. However, a large amount of CO_2_ evolution was identified (Fig. 5a) above 4.3 V, suggesting the chemical decomposition of Li_2_CO_3_ by the electrolyte decomposition byproducts at high potential. This result is consistent with Freiberg’s work^41^. The ratio e^−^/CO_2_ is 2.20, in good agreement with the literature^41^ but it does not mean that Li_2_CO_3_ is electro-oxidized via a 2-e process. Instead, the electrolyte decomposition is likely to produce active H^+^ at a ratio e^−^/H+ of 1 and the H^+^ reacts with Li_2_CO_3_ to release CO_2_ at a ratio H+/CO_2_ of 2^41^. Therefore, here the ratio e^−^/CO_2_ is just used for comparison between these four cells. Here, the chemical pathway does not take place until a high potential over 4.3 V when the electrolyte was electro-oxidized first. For comparison, the CO_2_ evolution in the charging process starts from ~3.9 V (Figs. 1–3), which is too low to electro-oxidize the electrolyte. Therefore, the Li_2_CO_3_ decomposition at low potential <3.9 V is dominated by the electrochemical pathway, instead of by a chemical pathway induced by electrolyte degradation by-products.

On the contrary, the same experiment but with LiTFSI-tetraglyme electrolyte shows little CO_2_ evolution (Fig. 5b), which makes a sharp comparison to the LiPF_6_-EC/EMC electrolyte. In LiTFSI-tetraglyme electrolyte. CO_2_ evolved from 4.3 V, indicating the decomposition of electrolytes at high potential could induce the chemical decomposition of Li_2_CO_3_. However, the ratio e^−^/CO_2_ is 12.29, much higher than that in LiPF_6_-EC/EMC. In addition, the total amount of CO_2_ is as low as 0.4 μmol, <2% of the CO_2_ evolution in the Super P-Li_2_CO_3_ electrode, Fig. 1a. This distinct comparison indicates that the reaction mechanisms of Li_2_CO_3_ decomposition are completely different in LiPF_6_-EC/EMC and LiTFSI-tetraglyme electrolytes. The chemical pathway proposed by Freiberg is not applicable for tetraglyme-based electrolytes. The difference is due to either the Li salts or the solvents.

To find out whether the Li salts or the solvents make the difference, the salts were exchanged in these electrolytes and LiTFSI-EC/EMC and LiFP_6_-tetraglyme were used, Fig. 5c and d. Interestingly, the LiPF_6_-tetraglyme shows much CO_2_ evolution similar to LiPF_6_-EC/EMC. Meanwhile, LiTFSI-EC/EMC shows little CO_2_ evolution similar to LiTFSI-tetraglyme. The cells using LiPF_6_ salt show a large amount of CO_2_ evolution. The electrolytes used in this work were all dried and the water concentrations were below 4 ppm (Karl-Fischer titration). Therefore, this chemical mechanism could be due to the LiPF_6_ salt itself or its impurities because LiPF_6_ is difficult to purify^53^. Freiberg et al. also proposed that the chemical pathway involving LiPF_6_ salt^41^. In the rest of this work, we used LiTFSI in tetraglyme as the electrolyte, thus excluding any possible effects of LiPF_6_ on our results. As little CO_2_ was evolved in the Celgard-separated experiment with LiTFSI-based electrolytes, our results provide evidence that Li_2_CO_3_ decomposition in LiTFSI-tetraglyme electrolytes is dominated by an electrochemical rather than chemical process.

We propose the following reactions for the electrochemical decomposition of Li_2_CO_3_ on carbon substrates:

5 $${{{{{{\rm{Li}}}}}}}_{2}{{{{{{\rm{CO}}}}}}}_{3}-2{{{{{{\rm{e}}}}}}}^{-}\to {{{{{{\rm{CO}}}}}}}_{2}+2{{{{{{\rm{Li}}}}}}}^{+}+1/{2}{\,\!}^{1}{{{{{{\rm{O}}}}}}}_{2}$$

6 $$(1-{{{{{\rm{x}}}}}}/2){\,\!}^{1}{{{{{\rm{O}}}}}}_{2}+{{{{{\rm{C}}}}}}\to (1-{{{{{\rm{x}}}}}}){{{{{{\rm{CO}}}}}}}_{2}+{{{{{\rm{x}}}}}}\,{{{{{\rm{CO}}}}}}$$

7 $${\,\!}^{1}{{{{{\rm{O}}}}}}_{2}+{{{{{\rm{electrolyte}}}}}}\to {{{{{{\rm{CO}}}}}}}_{2}+{{{{{\rm{CO}}}}}}+{{{{{\rm{byproducts}}}}}}$$

**Fig. 6:**
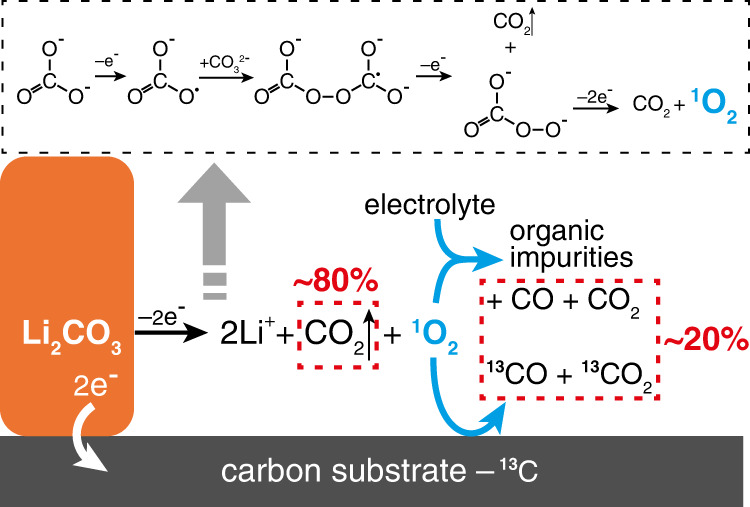
Schematics of the Li_2_CO_3_ decomposition on the charging process. Li_2_CO_3_ is electro-oxidized to form CO_2_ and singlet O_2_ (^1^O_2_). A possible pathway is proposed in the dash line box. ^1^O_2_ is highly reactive and it attacks the electrolyte and the carbon substrate to form carbon monoxide and carbon dioxide, which contribute to ~20% of the overall gas evolution.

Li_2_CO_3_ is oxidized at high potentials to form CO_2_ and aggressive ^1^O_2_ (Eq. 5), Fig. 6. ^1^O_2_ attacks carbon substrate (Eq. 6) and electrolyte (Eq. 7) simultaneously like incomplete combustion reactions, leading to further side reactions forming CO_2_ and CO. It should be noted here, Eq. (7) is just a schematics equation instead of a proper stoichiometric equation for an elementary reaction because we do not completely know all byproducts from electrolyte decomposition. The exact ratio CO_2_/CO depends on the ratio between formed ^1^O_2_, the carbon substrate/electrolyte, namely the electrode composition, but not on the cell potentials. Although the carbon substrate is eventually oxidized to CO_2_ and CO, the carbon does not directly participate in the electro-oxidation of Li_2_CO_3_ as has been proposed in the literature^28^. As we mentioned above, the Eq. (2) could be recognized as a combination of Eq. (5) and Eq. (6) when x is equal to zero in Eq. (6). In this case, the reactive intermediate ^1^O_2_ reacts with carbon to produce only CO_2_ instead of a mixture of CO_2_ and CO. However, our chemical reaction between ^1^O_2_ and ^13^C shows both ^13^CO_2_ and ^13^CO evolution (Supplementary Fig. 11). If a highly reactive intermediate other than ^1^O_2_ forms in the Li_2_CO_3_ decomposition and rapidly attacks carbon to just CO_2_, Eq. (2) could establish and contribute to a parallel pathway with Eq. (1) in Li_2_CO_3_ decomposition. However, there is so far no evidence of such a highly reactive intermediate.

Overall, during the charging process of the cell with a Li_2_CO_3_-carbon composite electrode in LiTFSI-tetraglyme electrolyte, Li_2_CO_3_ decomposition contributes the major CO_2_ evolution (~80%), and the carbon substrate and electrolyte decomposition share the rest ~20% of gas evolution. Therefore, in the charging process of Li-air batteries and Li-CO_2_ batteries, the decomposition of carbon substrate and the electrolyte is inevitable and contributes to a significant amount of CO_2_ and CO unless the ^1^O_2_ can be suppressed.

### Implications for batteries

Although Li_2_CO_3_ can be decomposed during the charging process, it is accompanied by severe side reactions by ^1^O_2_ (~20% of the total gas evolution) and leads to deterioration of cell performance. Here, we focus on Li-air and Li-CO_2_ batteries. In Li-air batteries, Li_2_CO_3_ is a major by-product and it passivates the electrode surface and kills the cells, which brings us to a dilemma. On one hand, if Li_2_CO_3_ is not decomposed during the charging process, Li_2_CO_3_ will rapidly accumulate during cell cycling and thus completely passivate the electrode. On the other hand, if Li_2_CO_3_ is decomposed during the charging process, it results in more side-reactions and thus accelerates cell deterioration. This effect is more significant in Li-CO_2_ batteries because Li_2_CO_3_ and carbon form are supposed to be the main discharge products. Therefore, a reversible decomposition of Li_2_CO_3_ and carbon is desired in the following charging process^16,17^. However, during the charging process, the discharge reaction cannot be completely reversed because the carbon does not directly participate in the Li_2_CO_3_ decomposition reactions. The carbon substrate is oxidized by the ^1^O_2_ intermediate to CO_2_ and CO. Meanwhile, the aggressive ^1^O_2_ attacks the electrolyte, leading to the depletion of the electrolyte and consequently cell failure. If the electrolytes and salts in the cells are sufficiently stable, e.g., solid-state electrolyte, the molten-salt electrolyte of LiNO_3_-KNO_3_, etc, the side-reactions with electrolyte could be avoided and ^1^O_2_ quenches to ^3^O_2_. Recently, Zhang’s group observed the O_2_ evolution when charging Li_2_CO_3_ in the cell using a solid-state electrolyte^54^. Due to the stability of inorganic solid-state electrolyte, ^1^O_2_ had nothing to attack and eventually was quenched to ^3^O_2_. However, even in this case, the ^1^O_2_ would oxidize the carbon substrate and thus the cell deterioration cannot be completely inhibited.

Therefore, the key to pursuing a better charging process is to design a catalyst that suppresses the formation of ^1^O_2_ rather than simply facilitates the kinetics of Li_2_CO_3_ decomposition and thus decreases the overpotentials. For instance, some redox mediators (RM) with moderate O_2_ binding energy could be applied to inhibit forming ^1^O_2_ and encourage ^3^O_2_ evolution by replacing the ^1^O_2_ precursor (e.g. CO_4_^2−^) with a low-energy RM-involved intermediate, just like the way 2,5-di-tert-butyl-1,4-benzoquinone does to O_2_^−^^55^. Alternatively, some solid catalysts with suitable O_2_ binding energy could bind ^1^O_2_ precursor (the key reaction intermediator of Li_2_CO_3_ decomposition) in order to lower the energy and stabilize the precursor before it transforms to ^1^O_2_. The ratio of ^1^O_2_ in the final product will decrease when the energy of the ^1^O_2_ precursor decreases.

Very recently, Hu’s group used *operando* electron paramagnetic resonance to show that Co_3_O_4_ inhibited the ^1^O_2_ formation during the charging process of Li-O_2_ cells^56^. Here, we added some Co_3_O_4_ nanoparticles to the Li_2_CO_3_-Super P composite electrode and then charged the electrode. As shown in Supplementary Fig. 16a, some O_2_ was identified during the charging process. While the control experiment of charging the Co_3_O_4_ electrode itself without Li_2_CO_3_ exhibits no O_2_ evolution, Supplementary Fig. 16b. This result confirms that the Co_3_O_4_ catalyst successfully interacts with the precursor of forming ^1^O_2_ during the Li_2_CO_3_ decomposition and thus suppresses ^1^O_2_ formation. On the other hand, the CO evolution with Co_3_O_4_ is only half of that without Co_3_O_4_, whereas similar CO_2_ evolutions were identified in both experiments. Because CO is from side-reactions of ^1^O_2_ attacks electrolyte/carbon substrate, decreased CO evolution confirms that ^1^O_2_ formation was partially inhibited and thus fewer side-reactions of ^1^O_2_ attacking the carbon substrate and the electrolyte were detected than the counterpart without Co_3_O_4_, Supplementary Fig. 17. Our results are in good agreement with Hu’s group results^55^. Although the ^1^O_2_ formation and CO evolution cannot be completely inhibited, the Co_3_O_4_ does make some effects by stabilizing the reaction intermediates of forming ^1^O_2_ and thus encouraging the evolution of ^3^O_2_. This example confirms the feasibility of this strategy to promote the Li_2_CO_3_ decomposition with less parasitic ^1^O_2_, however further studies are needed to look for more effective catalysts to avoid the side-reaction caused by ^1^O_2_.

To explore the decomposition mechanisms of the Li_2_CO_3_ and clarify the role of carbon substrate in the charging process of Li_2_CO_3_-carbon composite electrodes in LiTFSI-tetraglyme electrolyte, we did a set of in situ DEM-GC experiments with the ^13^C isotope-labeled composite electrodes to systematically isolate each component of the cell. The gas evolution during the charging process, including CO, CO_2_, ^13^CO, ^13^CO_2_, and O_2_ was quantified. Li_2_CO_3_ decomposed to release CO_2_ at an onset potential of 3.8 V mainly via an electrochemical mechanism. The chemical mechanism of Li_2_CO_3_ decomposition in literature could take place in the presence of LiPF_6_ due to LiPF_6_ itself or its impurities. Carbon substrate did not directly participate in the decomposition of Li_2_CO_3_, that is to say, carbon did not react with Li_2_CO_3_ in a single step to form CO_2_ as desired. On the contrary, this process is a multistep reaction. In the first step, Li_2_CO_3_ was oxidized to CO_2_ and ^1^O_2_. Then, the ^1^O_2_ simultaneously oxidized the carbon substrate and electrolyte to form CO_2_ and CO as gaseous side products. Approximately 80% of the net/cumulative CO_2_ evolution is contributed by the Li_2_CO_3_ decomposition and the rest ~20% is contributed by the decomposition of the carbon substrate and the electrolyte, which cannot be ignored in batteries, particularly in Li-CO_2_ batteries. In this work, we clarify the reaction mechanisms of the Li_2_CO_3_ decomposition during the charging process and exhibit the role of carbon in this process. This finding establishes a detailed picture of the decomposition pathways of Li_2_CO_3_ which enables strategy for the design of highly efficient cathode catalysts for Li-air and Li-CO_2_ batteries.

## Methods

### Materials

Lithium carbonate (Li_2_CO_3_), potassium superoxide (KO_2_), lithium bis(trifluoromethane)sulfonamide (LiTFSI), 9,10-dimethylanthracene (DMA), and Co_3_O_4_ were purchased from Sigma-Aldrich. Li_2_^13^CO_3_ and ^13^C were purchased from the Cambridge Isotope Ltd. Tetraethylene glycol dimethyl ether (tetraglyme), ethylene carbonate (EC), and methyl ethyl carbonate (EMC) were purchased from the TCI Chemical. Tetraglyme was distilled under vacuum and dried with activated molecular sieves (4 Å). Lithium hexafluorophosphate (LiPF_6_), ferrous sulfate (FeSO_4_), phosphoric acid (H_3_PO_4_), acetic acid, and hydrogen peroxide (H_2_O_2_) were purchased from Aladdin. Lithium iron phosphate (LFP) was purchased from Shenzhen Betterui New Materials Group Co., Ltd. Dimethyl sulfoxide-d_6_ (DMSO-d_6_) and 18-crown-6 were purchased from the Shanghai Yuanye Bio-Technology. Argon (N5 grade) and10 % Ar-O_2_ (N5 grade) were obtained from Nanjing Special Gas Ltd. Polytetrafluoroethylene emulsion (PTFE) was purchased from Innochem. Celgard separator (25 μm thickness, Celgard), glass fiber separator (GF/F, Whatman), and Super P carbon (Timcal) were purchased from Duoduo Chemical Technology Co. Ltd.

### Preparation of the composite electrodes

The blank carbon electrode, Li_2_CO_3_-carbon composite, and Li_2_CO_3_-Super P-Co_3_O_4_ composite (1:1:0.5) electrodes were prepared as described in literature^9,28,56^. Briefly, a certain amount of Super P, Li_2_CO_3_, and Super P carbon or Li_2_CO_3_, Super P and Co_3_O_4_, and PTFE were mixed and the mixture was ball milled overnight. The mass ratio of active material and binder PTFE is 10:1. A certain amount of well-mixed powder was weighed and absolute ethanol was added to obtain a slurry. The slurry was cast onto pre-washed stainless steel (SS) mesh (100 mesh) and the electrodes were dried under vacuum at 120 °C overnight. The mass loading is 5 mg per electrode. For instance, a Super P- Li_2_CO_3_ (1:1) composite electrode contains 2.27 mg Super P, 0.46 mg PTFE, and 2.27 mg Li_2_CO_3_ (equivalent to 30.7 μmol). For the ^13^C-isotope-labeled electrodes, the Li_2_CO_3_ and the Super P were replaced with the Li_2_^13^CO_3_ and ^13^C carbon, respectively. The ^13^C-isotope-labeled composite is only obtained by grinding because the ^13^C-isotope-labeled substance is too expensive.

### Preparation of the LFP electrode

80 mg LFP powder, 10 mg Super P, and 100 mg PTFE suspension (10%) were prepared. Firstly, 80 mg LFP and Super P were ground in a mortar to ensure that the LFP and Super P were evenly mixed. 0.1 mL of ethanol was dropped into the mixed powder to wet it and then the PTFE suspension was added to the mixed powder. After LFP-Super P and PTFE were mixed evenly, it was rolled several times with a roller. The final thickness of the electrode is 0.8 mm. The LFP electrode was punched into 12–25 mm diameter and then soaked in a solution (7.2 mL 30% H_2_O_2_ and 3 mL acetic acid in 500 mL H_2_O) for 30 min to precharge the LFP. The precharged LFP electrodes were rinsed with water five times to completely remove the residual H_2_O_2_ and acetic acid. Finally, the treated LFP electrodes were dried in a vacuum oven overnight and transferred to a glove box for later use.

### Potential calculation

Ag wire was used as a pseudo-reference electrode in a three-electrode cell. We immersed the Ag wire in 1 M LITFSI tetraglyme electrolyte with O_2_ to stabilize the potential of the Ag wire. A three-electrode cell with a LFP working electrode was charged and discharged in 1 M LiTFSI-tetraglyme using this Ag wire as the reference electrode. The equilibrium potential of LFP was 0.5 V versus Ag wire. Considering the potential of LFP versus Li^+^/Li is 3.45 V, the potential of the Ag wire is 2.95 V versus Li^+^/Li. Therefore, the potentials versus Li^+^/Li of the experiments in 1 M LiTFSI-tetraglyme could be calculated. All potentials in this manuscript are versus Li^+^/Li without further notice.

### DEMS-GC setup

A differential electrochemical mass spectrometer (DEMS, Prima BT, Thermo Scientific Ltd.) was coupled with a gas chromatograph (GC, Hope Ltd.) in parallel (Supplementary Fig. 1). The GC is equipped with a TCD and an FID detector (including a CO_2_/CO converter). The DEMS cell is based on a customized Swagelok design. It was assembled and charged/discharged in the Ar-filled glove box. The Ar carrier gas carried the evolved gas in the cell into DEMS and GC simultaneously. The typical flow rate of Ag carrier gas is 0.5 mL min^−1^ and the sweep rate for LSV is 0.05 mV s^−1^. The time resolutions of DEMS and GC are 10 seconds and 5 min, respectively. The general gas evolution in chemical reactions was examined by the mass spectrometer (MS, Prima BT) itself.

### Experiments of Li_2_CO_3_ with 1000/5000 ppm H_2_O in 1 M LiTFSI-tetraglyme electrolyte

Firstly, 1000 ppm and 5000 ppm H_2_O were added to the 1 M LiTFSI-tetraglyme electrolyte, respectively and the water concentration in the electrolyte was quantified by a Karl Fischer titrator (Mettler Toledo). Then, the gas analysis was conducted in a MS. Generally, 100 mg commercial Li_2_CO_3_ was added to a vial that was connected to a MS. After passing in the carrier gas for a while to obtain a steady baseline, 1 mL electrolyte with H_2_O was added to the vial and it was stirred for several hours. The gas evolution was quantified by a MS.

### Quantification of gas evolution during the charging process

The composite electrodes were charged in a homemade differential electrochemical mass spectrometry cell. A piece of precharged LiFePO_4_ served as the counter electrode and a piece of silver wire served as the reference electrode. 0.2 mL of 1 M LiTFSI-tetraglyme served as the electrolyte. The water content of the electrolyte was <4 ppm tested by Karl Fischer moisture titrator. To study the impact of the potential on the charging process, the composite electrodes were charged using linear sweep voltammetry (LSV) with a sweep rate of 0.05 mV s^−1^. Due to the stability window (4.8 V) of the tetraglyme-based electrolyte, the cell potential was cut off at 4.7 V. Argon was served as the carrier gas and its flow is 0.5 ml/min. The gas evolution was examined by a magnet sector mass spectrometer (Prima BT, Thermo Scientific Ltd.).

For the experiments for the chemical route, the Li_2_CO_3_ was deliberately separated from the Super P-PTFE composite electrolyte to allow the electrolyte decomposition byproduct to decompose Li_2_CO_3_ chemically. Twenty milligrams of commercial Li_2_CO_3_ was weighed and dusted to the top of the separator. A piece of Celgard separator (25 μm thickness) was stacked on the Li_2_CO_3_ and then a Super P-PTFE composite electrode is stacked on the Celgard. The composite electrode was electronically isolated from Li_2_CO_3_ by the Celgard separator. For the 1 M LiTFSI-tetraglyme electrolyte, the cell was charged to 4.7 V and the gas evolution was recorded. For the control experiment with EC/EMC electrolyte, due to the volatile electrolyte, the cell was not purging with Ar carrier gas during the entire charging process. The gas evolution in the headspace was purged with Ar at the end of the charging process to quantify the CO_2_ evolution during the entire charging process. A homemade in-line cold trap (−25 °C) was applied to condense the evaporated EMC to minimize the background noise caused by EMC.

### Quantification of CO

The mass-to-charge (*m/z*) ratio of 28 could be contributed by CO, N_2_, and the fragment of CO_2_. To exclude the contribution of the N_2_, the gas inlet of the DEMS was placed inside an Ar-filled glovebox as shown in Supplementary Fig. 1 and the DEMS experiments were conducted inside the glove box at 25 ± 2 °C to minimize the impact of N_2_ leakage. The *mz* = 28 fragment from CO_2_ was calibrated with a calibration gas of 1% CO_2_–99% Ar using DEMS. The intensity of the *mz* = 28 signal is 6.5% of the *mz* = 44 signal, which is subtracted from the concentration of CO in the calculation. This ratio(*mz* = 28/*mz* = 44) depends on many factors, such as electron voltage, emission current of the filaments, etc. Therefore, this ratio needs to be calibrated for individual MS. A calibration gas of 0.1% CO–99.9% Ar was used for calibration.

The CO evolution during the charging process was parallelly quantified by a GC with a TCD and a FID detector (including a CO_2_/CO converter). A FID detector was used to quantify the CO and 1000 ppm CO equals an area of 130 mVs in GC. Thus we could use these results as a reference and qualify the amount of CO in all experiments.

### Quantification of the solid byproducts

The solid Li_2_CO_3_ and organic carbon in the composite electrodes were quantified as reported previously^57^. An electrode was placed in a vial that is connected to a MS and 0.5 ml of H_3_PO_4_ (2 M) was injected into the vial to react with Li_2_CO_3_ to form CO_2_. The CO_2_ evolution was quantified by MS. Afterward, Fenton solution (400 μL FeSO_4_ + 50 μL of H_2_O_2_ (30%)) was added to oxidize the organic carbonates to CO_2_. The released CO_2_ was again quantified by a MS.

### Isotope impurities in Li_2_^13^CO_3_ and ^13^C-carbon

A certain amount of Li_2_^13^CO_3_ reacts with H_3_PO_4_ solution in a vial that is connected to a MS. The gas evolution was quantified by MS and shown in Supplementary Fig. 5. 16.92 μmol of ^13^CO_2_ and 2.49 μmol ^12^CO_2_ are identified. Therefore, the ratio between ^12^CO_2_ and ^13^CO_2_ is 0.147/1. This result indicates that the commercial Li_2_^13^CO_3_ sample contains c.a. 15% of ^12^C-impurity.

The ^12^C impurity in the ^13^C is determined by a MS as well. The carbon was combusted under a O_2_ flow (0.5 mL min^−1^) in a quartz tube that was connected to a MS. The formed ^13^CO_2_ and ^12^CO_2_ are quantified and they are 82.42 μmol and 1.27 μmol, respectively. The ^13^C sample contains 1.5% of ^12^C as an isotope impurity.

### Identification of ^1^O_2_

30 mM DMA was added into 1 M LiTFSI-tetraglyme electrolyte as a molecular trap to singlet O_2_. The composite electrode was electrochemical oxidation by linear sweep voltammetry (LSV) with a voltage cutoff of 4.2 V. The electrolyte was extracted from all cell components using DMSO-d_6_ for further experiment. ^1^H-NMR spectra were recorded on a Bruker Avance III 300 MHz FT NMR spectrometer with autosampler (300.36 MHz, DMSO-d_6_).

We lack an effective way to quantify the ^1^O_2_ during charging to such a high potential of 4.7 V. On one hand, DMA decomposes above 4.2 V and thus we cannot use DMA to detect ^1^O_2_ at 4.7 V. However, there is only a small portion (<5%) of Li_2_CO_3_ decomposes below 4.2 V and the amount of ^1^O_2_ at this stage is low, which could not represent the overall reaction up to 4.7 V. For the aspect of energy, more ^1^O_2_ would form at a higher potential. On the other hand, the NMR is only a semi-quantitative technique. Thus probing DMA-O_2_ using NMR could only confirm the formation of ^1^O_2_ but it could not provide a reliable quantification of such a low amount of ^1^O_2_. The NMR of the electrolyte in a cell with Co_3_O_4_ is shown in Supplementary Fig. 18. The cell was charged by LSV to 4.2 V. The electrolyte was extracted from all cell components using DMSO-d_6_. The signals of DMAO_2_ in both samples are too weak to compare quantitatively.

### Chemical experiments between reactive oxygen species and carbon

The purpose of this ex situ chemical experiment is to identify the products of reactive oxygen species attacking ^13^C-carbon. We try to prove the feasibility that ^1^O_2_ attacks ^13^C-carbon to form ^13^CO_2_ and ^13^CO, which is detected during the charging process of the cell with Li_2_CO_3_. Meanwhile, our results show that superoxide species could not oxidize ^13^C-carbon to form ^13^CO_2_ and ^13^CO.

Singlet oxygen was obtained by the disproportionation of superoxide species, which is KO_2_ and Li^+^ here. Briefly, a varying amount of ^13^C-carbon and 22 mg KO_2_ powder was added to a vial that is connected to a MS before the solution is added into the vial. The carrier gas will be passed for a period to eliminate the residual gas in the vial. Then, 1 mL high concentration lithium salt electrolyte (4 M LiTFSI here) was injected into the vial to react with KO_2_ to disproportionate to produce ^1^O_2_. ^1^O_2_ immediately oxidized ^13^C-carbon to evolve ^13^CO_2_ and ^13^CO. The produced gases were detected by a MS. An excess amount of KO_2_ was used here to produce a large amount of ^1^O_2_. So far, we could not quantify the amount of ^1^O_2_ produced in this disproportionation reaction of KO_2_ and Li^+^. Here, the experiments were carried out without applying a potential, namely at “OCV”. The reactivity of carbon and the ratio of CO_2_/CO may change when voltage is applied. The impact of potential in the side-reaction of ^1^O_2_ and ^13^C is not the focus of this work. Also, when a high potential is applied to the carbon substrate, electrode decomposition will take place, which makes the situation complicated to decouple these factors. Here, we mainly focus on Li_2_CO_3_ electro-oxidation. Although carbon oxidation accompanies Li_2_CO_3_ decomposition, it only contributes to ~10% of the total CO_2_ evolution.

A control experiment of superoxide attacking ^13^C-carbon was carried out to make a comparison. Superoxide was obtained as previously reported^52^. In brief, firstly, argon was used to exclude the dissolved gas such as oxygen in the tetraglyme solvent. Then, 71 mg KO_2_ and 264 mg 18-crown-6 were added to 10 mL tetraglyme electrolyte and stirred for four hours to maximize the concentration of the O_2_^−^_(sol)_. The electrolyte was centrifuged and the supernatant also was injected with argon to get out the dissolved oxygen produced by the KO_2_ attack electrolyte for further experiment. Finally, varying amounts of ^13^C-carbon powder were added to a vial and 1 mL supernatant was injected into the vial for the source of superoxide. The produced gases were detected by a MS.

In the charging process of the cell, because Li_2_CO_3_ has ultralow ionic and electronic conductivities, its decomposition takes place at the ^13^C|Li_2_CO_3_ solid-solid interface (namely the contact points between ^13^C particles and Li_2_CO_3_ particles). Therefore, the decomposition product, ^1^O_2_, is just formed at this interface, which is very close to ^13^C, and thus it is easy to attack ^13^C. Therefore, the ratio between ^1^O_2_ and ^13^C is high. On the contrary, in the chemical experiments described above, ^1^O_2_ originates from the disproportionation of superoxide in the solution phase. Although ^13^C was added to the solution and stirring was applied, the fresh ^1^O_2_ forms from the disproportionation process in the electrolyte and it first attacks the electrolyte. Only a small portion of ^1^O_2_ would diffuse to the surface of the suspended ^13^C in the electrolyte and react with ^13^C. In addition, the ratio of releasing ^1^O_2_ from disproportionation is still unknown, which is highly likely below 10%. Freunberger et al show that more ^1^O_2_ forms during the charging process than in the discharging process involving disproportionation.

### Quenching ^1^O_2_

Once the ^1^O_2_ forms in the electrolyte, it is too late to stabilize it and it rapidly attacks the electrolyte and carbon substrate. Therefore, our strategy is to stabilize this intermediate/precursor which further produces singlet oxygen, rather than to quench the ^1^O_2_ after its formation in the electrolyte. In this case, we hope that Co_3_O_4_ binds the intermediate (namely the precursor of ^1^O_2_, e.g. CO_4_^2−^) into a complex such as Co_3_O_4_-CO_4_, before ^1^O_2_ is formed and dissolved in the electrolyte. Our experimental results show that Co_3_O_4_ makes some positive effects, but it is not good enough. If it works properly, the ratio(CO_2_/O_2_) should be 2 according to the equ 1a, but the ratio(CO_2_/O_2_)_Co3O4_ is 6.7, much higher than 2. It might be a feasible strategy to inhibit ^1^O_2_ formation but there are still lots of work that need to be done.

## Supplementary information

Supplementary Information File

## Data Availability

Source Data for Figs. 1–5 is provided with the paper. Extra data are available from the corresponding authors upon reasonable request. Source data are provided with this paper.

## Source data

Source Data
