# Supplementary material for: Oxidative decomposition mechanisms of lithium carbonate on carbon substrates in lithium battery chemistries
Source: Nat Commun. 2022 Aug 20;13:4908. doi: 10.1038/s41467-022-32557-w (PMC9392741; doi:10.1038/s41467-022-32557-w)
Supplement: Supplementary file 1 — Supplementary Information File [file 41467_2022_32557_MOESM1_ESM.pdf]

Supplementary Information for

Oxidative Decomposition Mechanisms of Lithium Carbonate on Carbon Substrates in Lithium Battery Chemistries

Authors: Deqing Cao[1+], Chuan Tan[1+], Yuhui Chen[1]*

Affiliations: [1]State Key Laboratory of Materials-Oriented Chemical Engineering, Nanjing Tech University, Nanjing, 211816, China

**Address correspondence to:* cheny@njtech.edu.cn

[+]Authors contributed equally to this work.

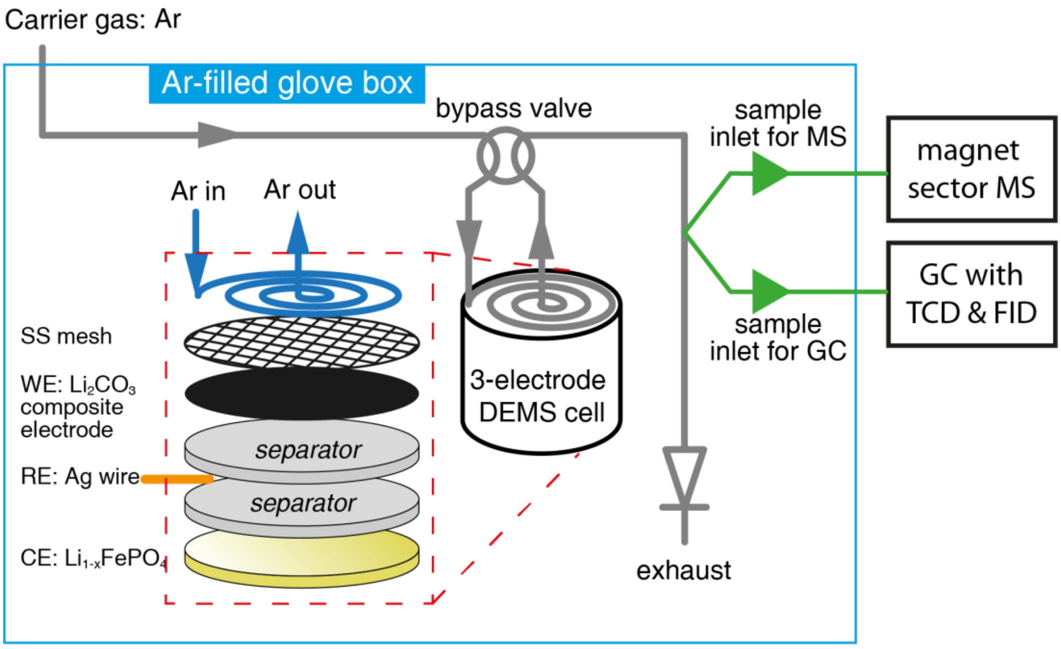

Figure S1. Schematics of the *in-situ* DEMS-GC system with a three-electrode cell inside an Ar-filled glovebox.

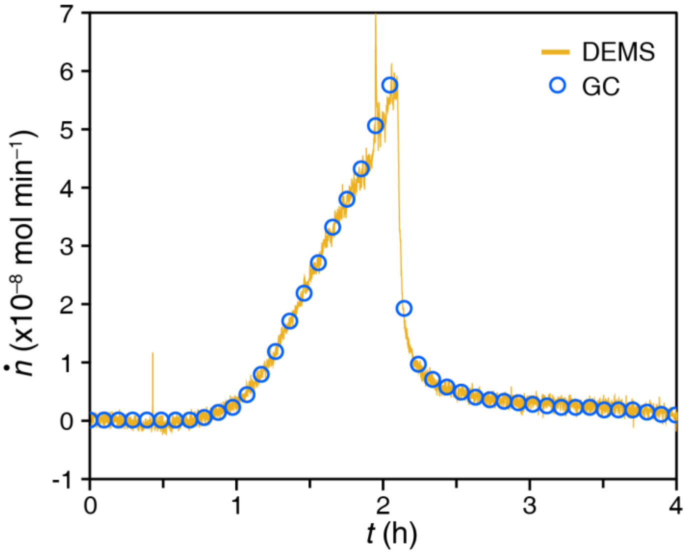

Figure S2. CO evolution recorded by DEMS (line) and GC (circles), which are consistent with each other.

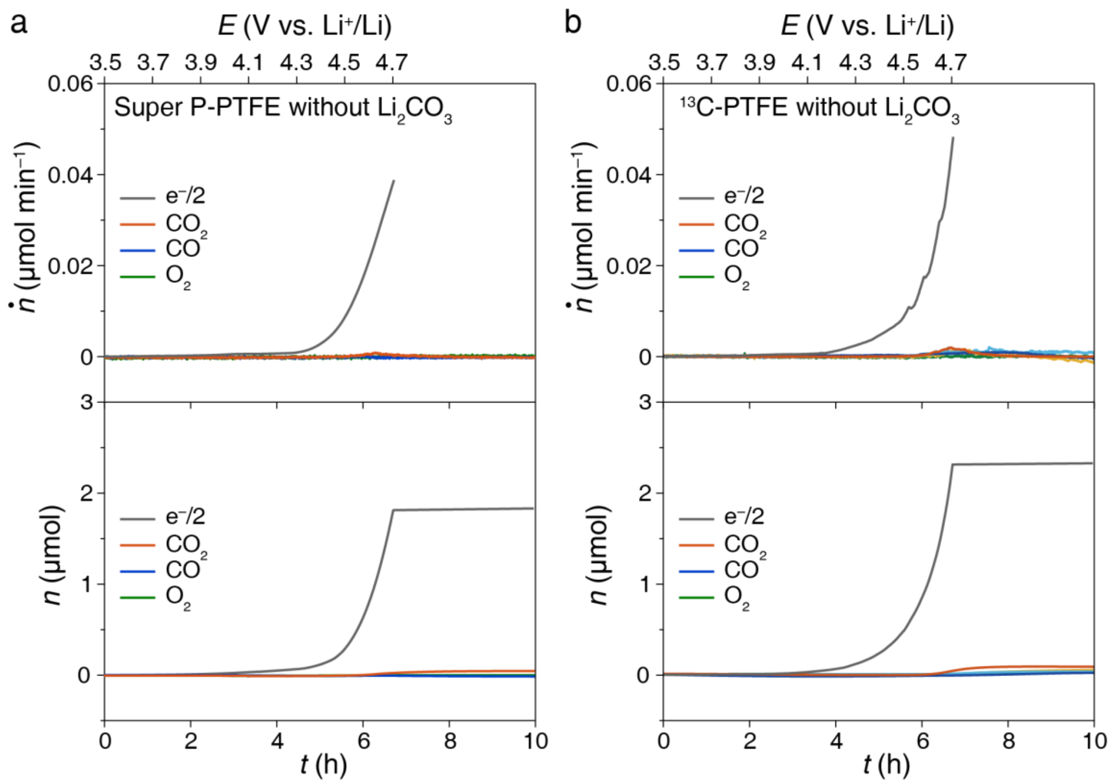

Figure S3. Gas evolution during the charging process of the cells using (a) Super P-PTFE (b) ^{13}C-PTFE composite electrodes in 1 M LiTFSI-tetraglyme. Ar flow rate: 0.5 mL min^{-1}. Sweep rate: 0.05 mV s^{-1}. The molar flux (top panel) of gas evolution was denoted as $\dot{n}$ and the charging current is translated to $\dot{n}_{electron}$ and the cumulative mole (bottom panel) of the gas was denoted as n. The blank carbon electrodes contain 2 mg of Super P and ^{13}C, respectively.

Table S1. The molar ratio between electrons and CO_2 evolution during the charging process of the cells using various electrodes.

electrode	***ratio*(e⁻/CO₂)**
Li_2CO_3-super P (1:1)	2.09
Li_2CO_3-super P (4:1)	2.18
$Li_2{}^{13}CO_3$-super P (1:1)	2.43
Li_2CO_3-^{13}C (2:1)	2.30
Li_2CO_3-^{13}C (1:1)	2.31
Li_2CO_3-^{13}C (1:2)	2.48
Li_2CO_3-^{13}C (1:4)	2.28

Table S2. Gas evolution during the charging process of the cells using Li_2CO_3-Super P composite electrodes with various ratios of Li_2CO_3/Super P.

**ratio(Li_2CO_3/SuperP)**	**n(CO_2)** /μmol	**n(CO)** /μmol	***ratio***$_{CO2/CO}$
**1:1**	21.6	3.06	7.06
**4:1**	10.8	1.10	9.82

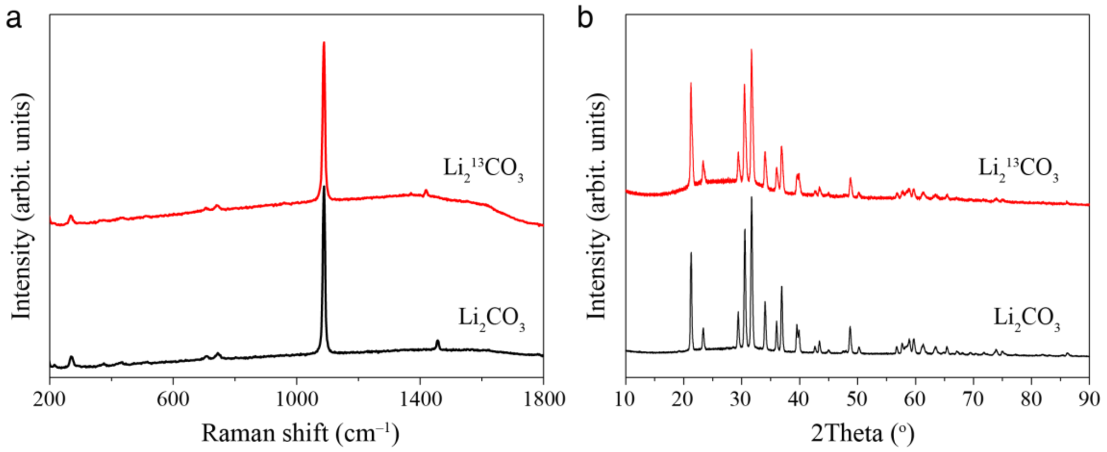

Figure S4. Characterization of $Li_2{}^{13}CO_3$ and Li_2CO_3. (a) Raman spectra and (b) XRD pattern of the $Li_2{}^{13}CO_3$ and Li_2CO_3.

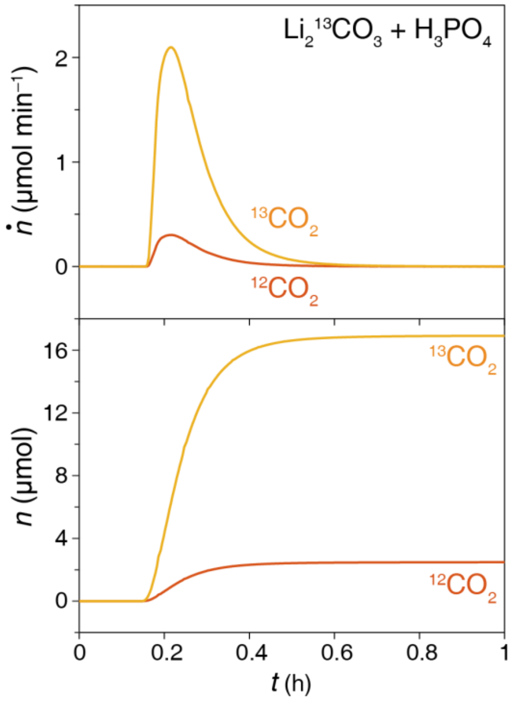

Figure S5. Quantification of ^{12}C-isotope impurities in $Li_2{}^{13}CO_3$. $^{13}CO_2$ and $^{12}CO_2$ evolution from the reaction between $Li_2{}^{13}CO_3$ and H_3PO_4. $^{12}CO_2$ evolved from $Li_2{}^{12}CO_3$ as the isotope impurity in $Li_2{}^{13}CO_3$.

Table S3. Gas evolution during the charging process of the cell using Li_2CO_3-^{13}C composite electrodes with various ratios of Li_2CO_3/^{13}C.

Li_2CO_3/^{13}C	CO_2/μmol	CO/μmol	$^{13}CO_2$/μmol	^{13}CO/μmol	***ratio*** $^{13}CO_2$/^{13}CO	***ratio***($^{13}CO_2$+^{13}CO/total gas)
2:1	5.71	0.64	0.42	0.34	1.25	10.6%
1:1	8.29	0.73	0.61	0.54	1.12	11.3%
1:2	8.37	0.26	0.37	0.49	0.76	9.1%
1:4	7.04	0.28	0.28	0.55	0.51	10.1%

Table S4. Gas evolution during the charging process of the cell using $Li_2{}^{13}CO_3$-Super P composite electrodes.

**n(CO_2)** /μmol	**n(CO)** /μmol	**n($^{13}CO_2$)** /μmol	**n(^{13}CO)** /μmol	***ratio*** **($^{13}CO_2$/total gas)**	***ratio*** **($^{13}CO_2/CO_2$)**	***ratio*** **($^{13}CO_2/(CO+CO_2)$)**
**0.79**	0.47	4.83	0	79%	6.1	3.8

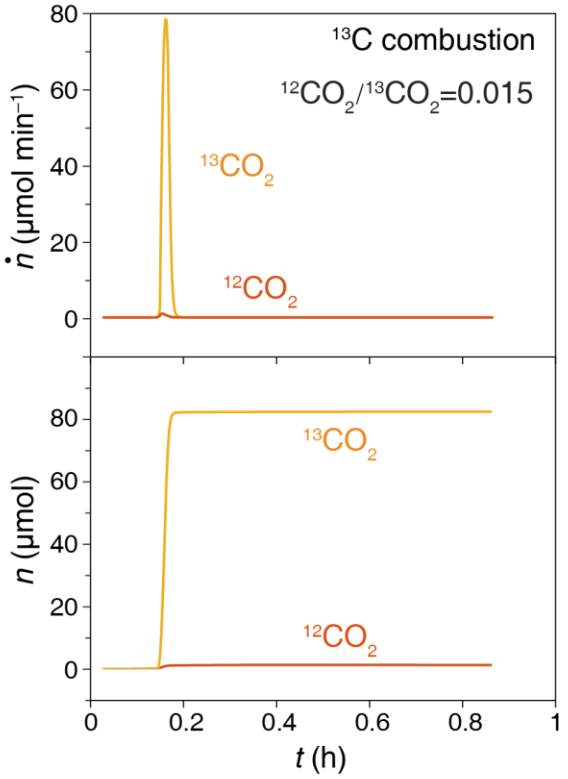

Figure S6. Quantification of ^{12}C-isotope impurities in ^{13}C-carbon. $^{13}CO_2$ and $^{12}CO_2$ evolution from the combustion reaction of ^{13}C-carbon under O_2. $^{12}CO_2$ is from the ^{12}C-carbon impurity in ^{13}C-carbon.

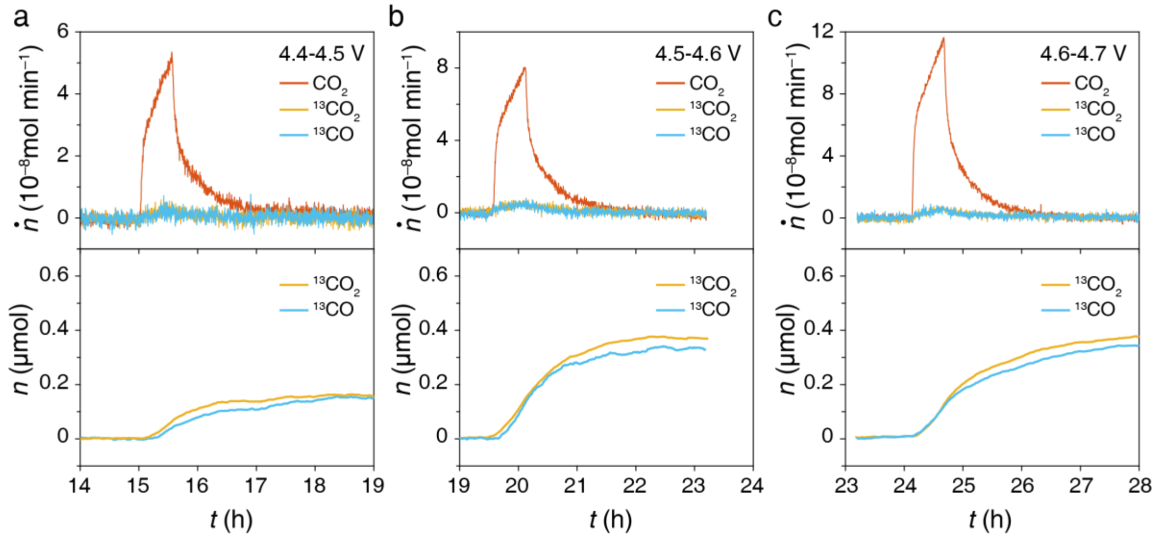

Figure S7. A supplementary figure to Figure 2 showing gas evolution of CO_2, $^{13}CO_2$, and ^{13}CO during the charging process of the cells using Li_2CO_3-^{13}C (1:1) composite electrode in 1 M LiTFSI-tetraglyme at (a) 4.4-4.5V, (b) 4.5-4.6V, and (c) 4.6-4.7V. All the potentials here are vs. Li^+/Li. The molar flux (top panel) of gas evolution was denoted as $\dot{n}$ and the charging current is translated to $\dot{n}_{electron}$ and the cumulative mole (bottom panel) of the gas was denoted as n.

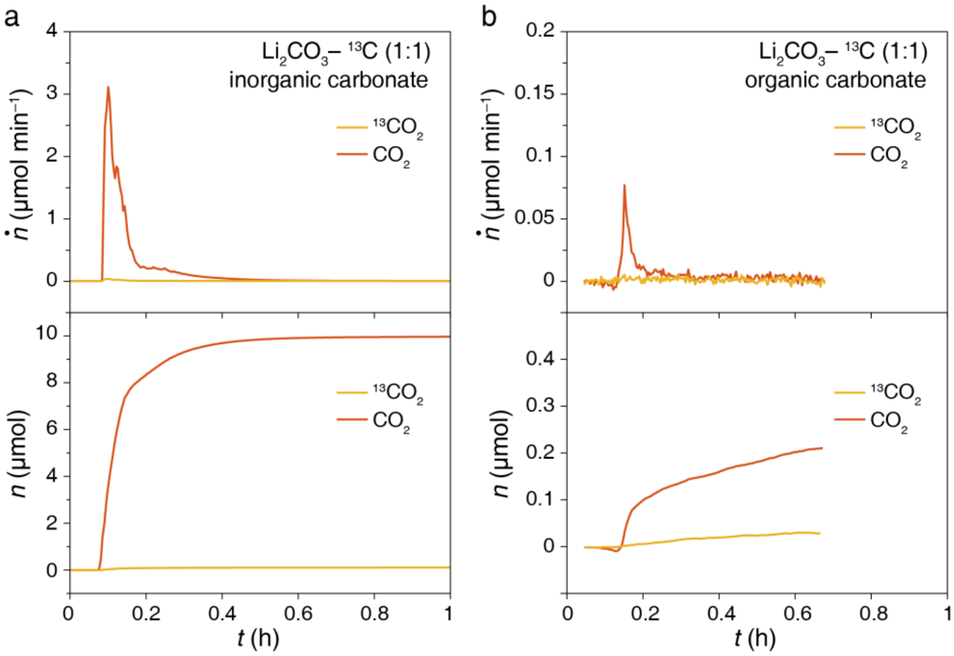

Figure S8. Quantification of (a) inorganic carbonate ($Li_2{}^{13}CO_3$/Li_2CO_3) and (b) organic carbonates byproducts in the Li_2CO_3-^{13}C (1:1) composite electrodes at the end of the charging process. The composite electrode reacts with H_3PO_4 and Fenton solution to quantify the inorganic carbonate and organic carbonate species, respectively.

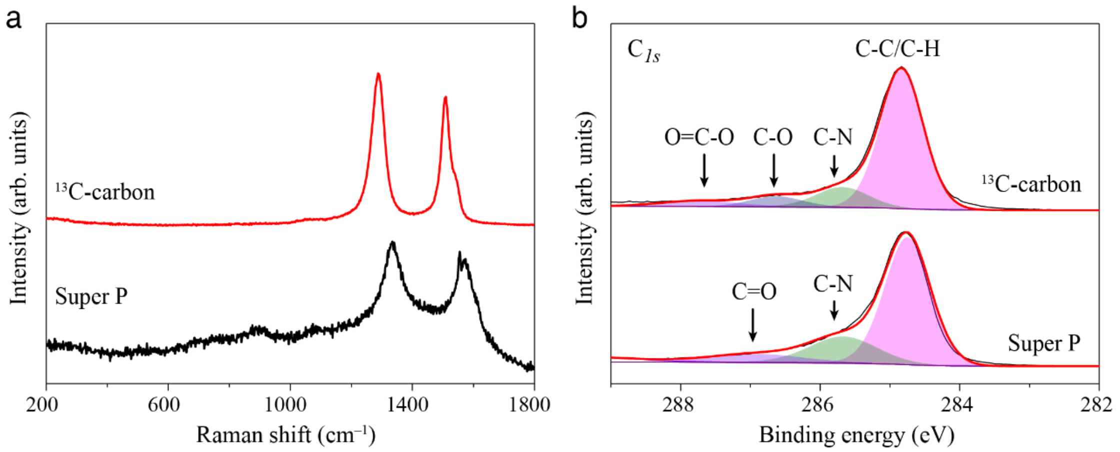

Figure S9. Characterization of ^{13}C-carbon and Super P carbon. (a) Raman spectra and (b) XPS spectra of ^{13}C-carbon and Super P.

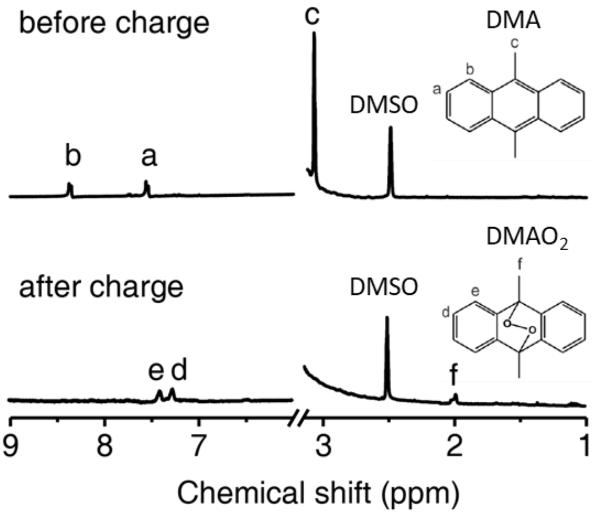

Figure S10. ^{1}H NMR of the electrolyte in the cell with Li_2CO_3-Super P (1:1) composite electrode before charging and at the end of the charging process. DMA was added into the electrolyte as a molecular trap to singlet O_2. $DMAO_2$ was identified at the end of the charging process.

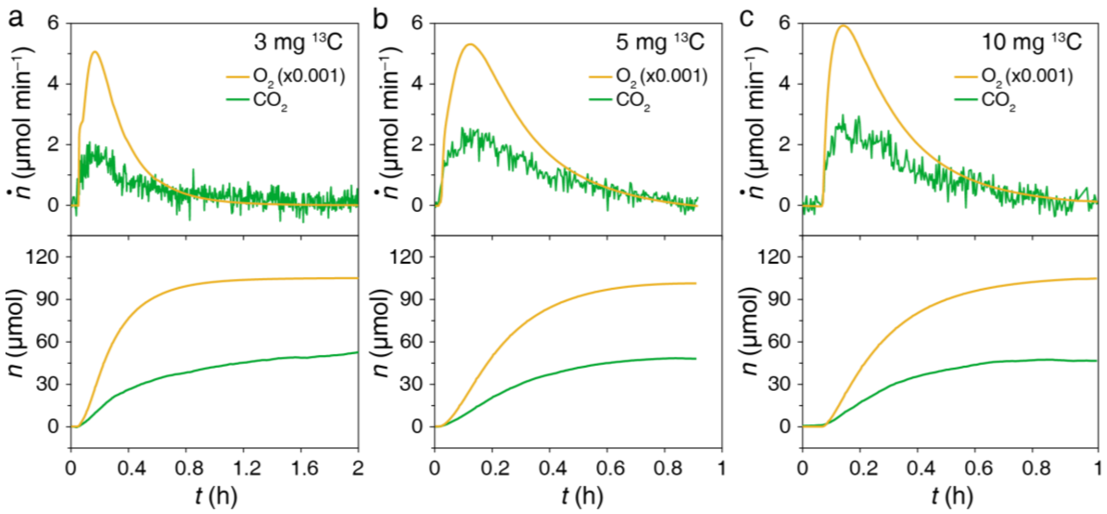

Figure S11 $^{12}CO_2$ and O_2 evolution of the chemical reaction of $^1O_2$ oxidizing ^{13}C in tetraglyme solution with the increasing amount of the ^{13}C-carbon. (a) 3 mg, (b) 5 mg, and (c) 10 mg ^{13}C was used.

Table S5. ^{13}CO and $^{13}CO_2$ evolution in the chemical reaction of $^1O_2$ oxidizing ^{13}C.

^{13}C	$^{13}CO_2$ /nmol	^{13}CO /nmol	***ratio***$_{^{13}CO_2/^{13}CO}$
3mg	39.4	17.1	2.3
5mg	35.4	16.9	2.1
10mg	20.7	13.0	1.6

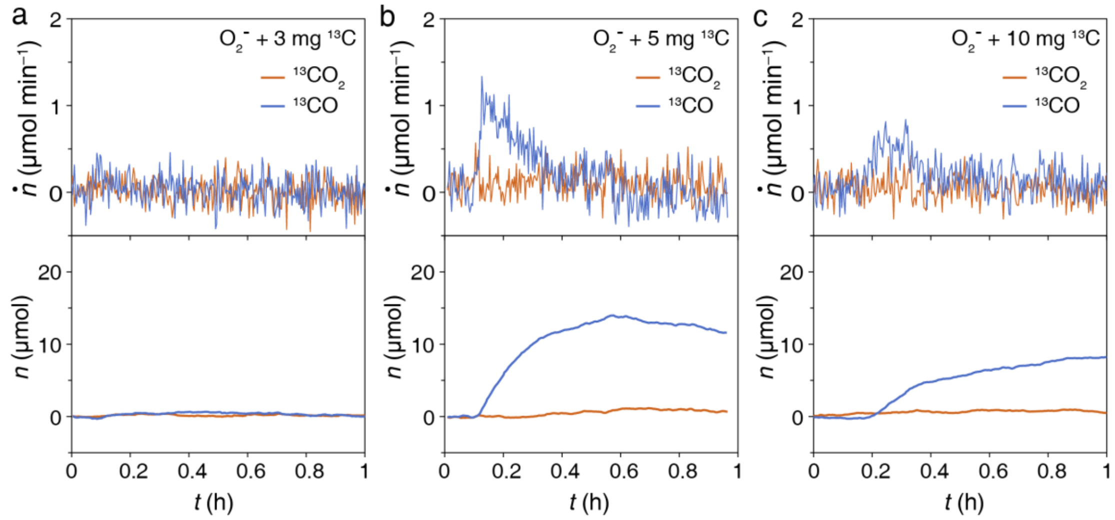

Figure S12. $^{13}CO_2$ and ^{13}CO evolution of the reaction between O_2^-(sol) and ^{13}C-carbon in tetraglyme solution with the increasing amount of the ^{13}C-carbon. (a) 3 mg, (b) 5 mg, and (c) 10 mg ^{13}C was used.

Table S6. ^{13}CO and $^{13}CO_2$ evolution during the charging process of the cell using $Li_2{}^{13}CO_3$-^{13}C (1:1) composite electrode.

CO_2 /μmol	CO /μmol	$^{13}CO_2$ /μmol	^{13}CO /μmol	***ratio***$_{CO2/CO}$	***Ratio*** $(CO_2+{}^{13}CO_2)/(CO+{}^{13}CO)$	***Ratio*** $(CO_2+CO/total\ gas)$
0.76	0.73	9.83	0.64	1.04	7.73	12.4%

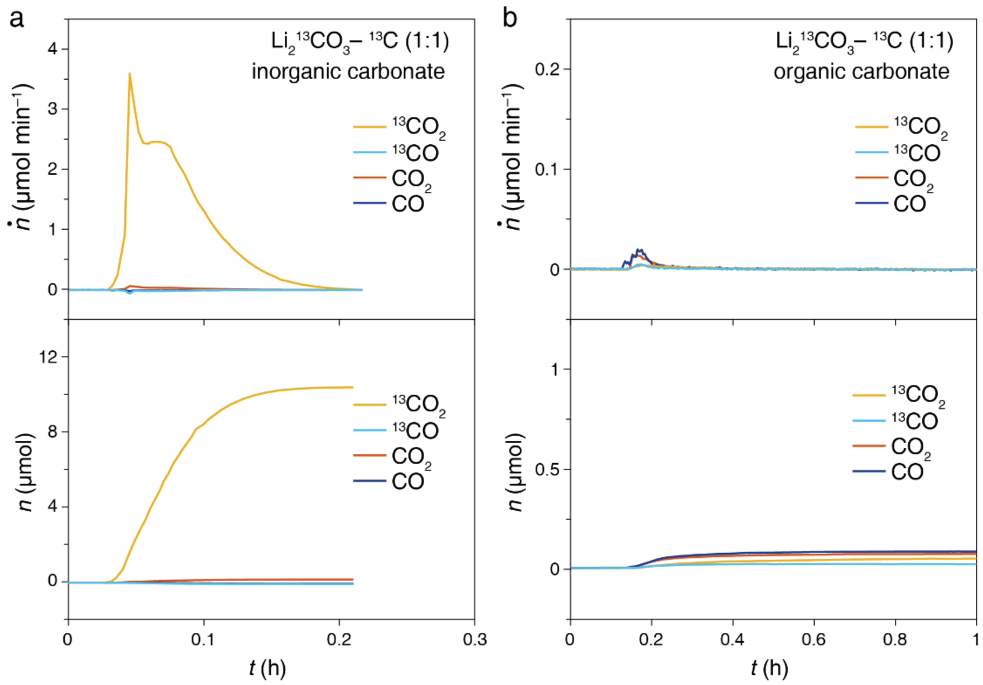

Figure S13. Quantification of (a) Li_2CO_3/$Li_2{}^{13}CO_3$ and (b) organic carbonates in the $Li_2{}^{13}CO_3$-^{13}C (1:1) composite electrodes at the end of the charging process. The composite electrode reacts with H_3PO_4 and Fenton solution to quantify the inorganic carbonate and organic carbonate species, respectively.

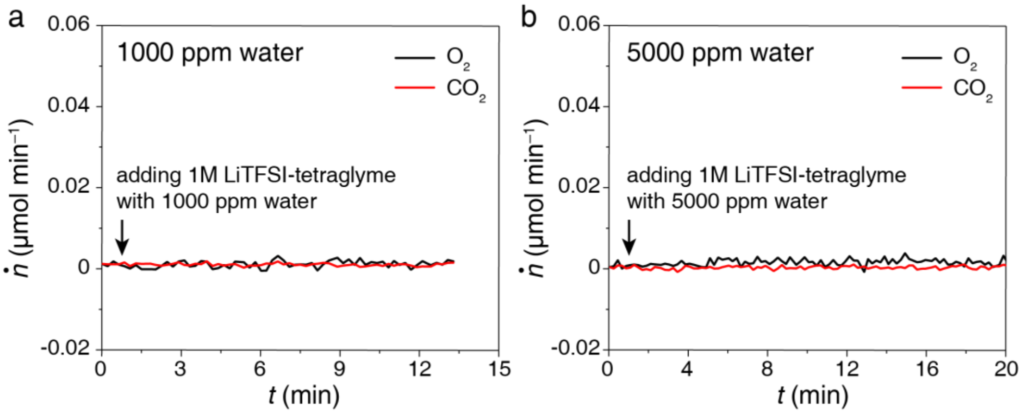

Figure S14 The side-reaction between water and electrolyte. Gas evolution of the reaction between commercial 100 mg Li_2CO_3 and 1 ml 1M LiTFSI- tetraglyme with (a) 1000 ppm and (b) 5000 ppm water. No CO_2 and O_2 evolution was identified.

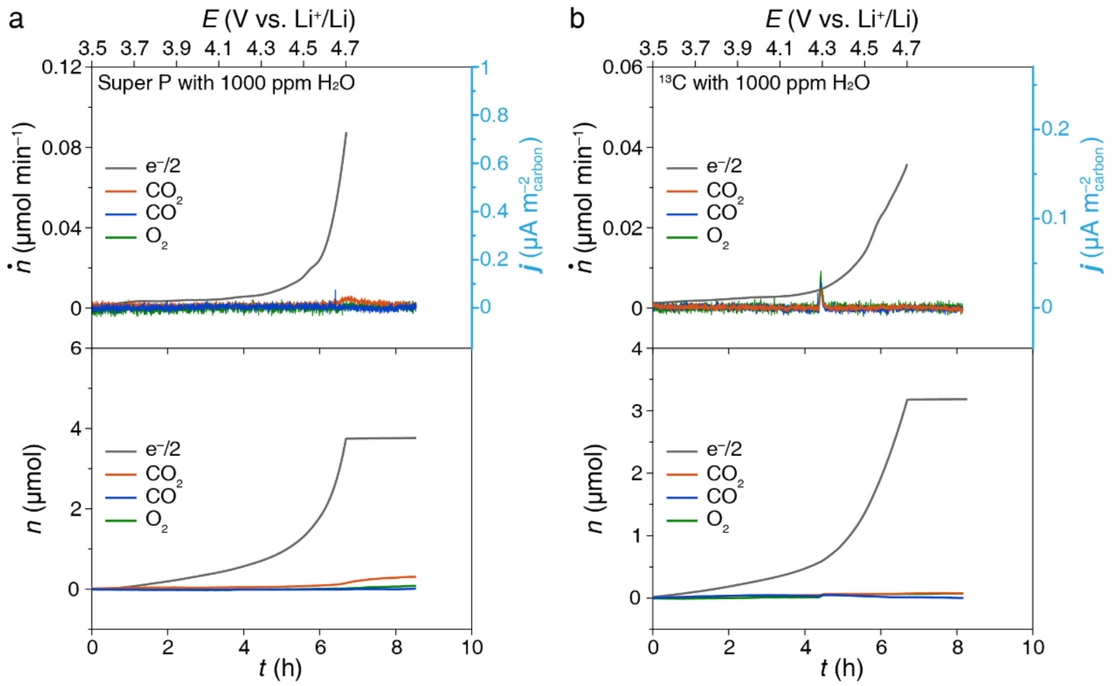

Figure S15 Gas evolution during the charging process of the cells using (a) Super P-PTFE and (b) ^{13}C-PTFE composite electrodes in 1 M LiTFSI-tetraglyme with 1000 ppm H_2O. Ar flow rate: 0.5 mL min^{-1}. Sweep rate: 0.05 mV s^{-1}. The molar flux (top panel) of gas evolution was denoted as $\dot{n}$ and the charging current is translated to $\dot{n}_{electron}$ and the cumulative mole (bottom panel) of the gas was denoted as n. The y-axis represents the anodic current density based on surface area of carbon in the composite electrode.

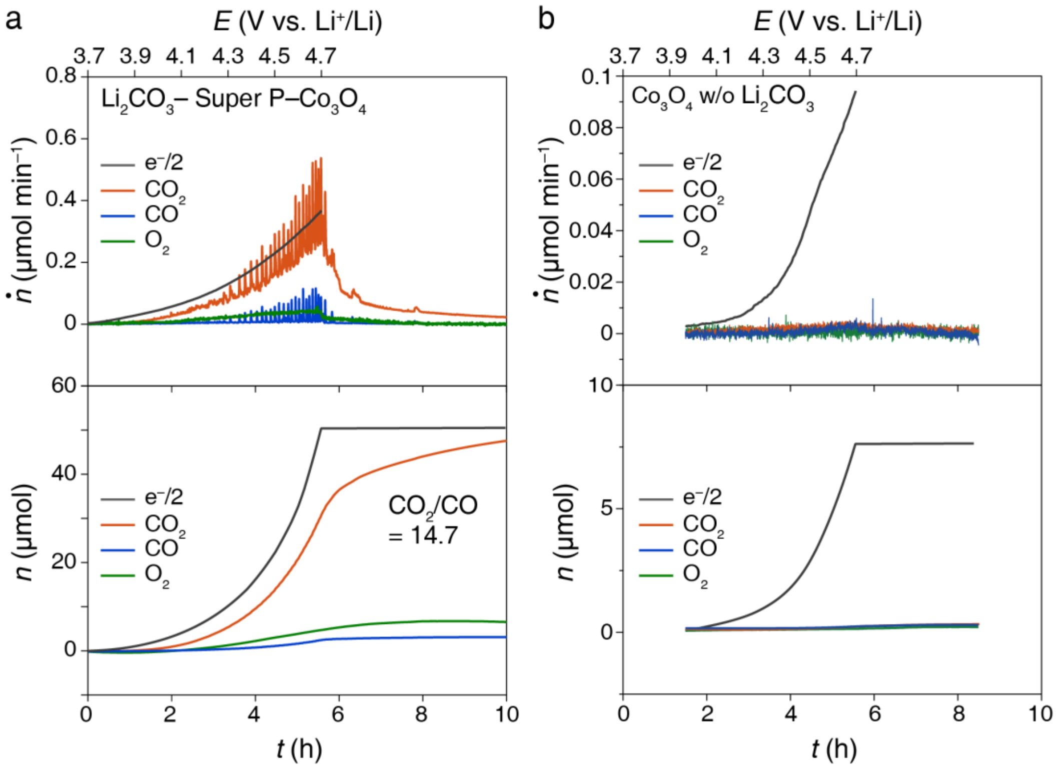

Figure S16. Gas evolution during the charging process of the cells using (a) Li_2CO_3-Super P-Co_3O_4 (1:1:0.5) and (b) Super P-Co_3O_4 (1:0.5) composite electrodes in 1 M LiTFSI-tetraglyme. Ar flow rate: 0.5 mL min^{-1}. Sweep rate: 0.05 mV s^{-1}. The molar flux (top panel) of gas evolution was denoted as $\dot{n}$ and the charging current is translated to $\dot{n}_{electron}$ and the cumulative mole (bottom panel) of the gas was denoted as n.

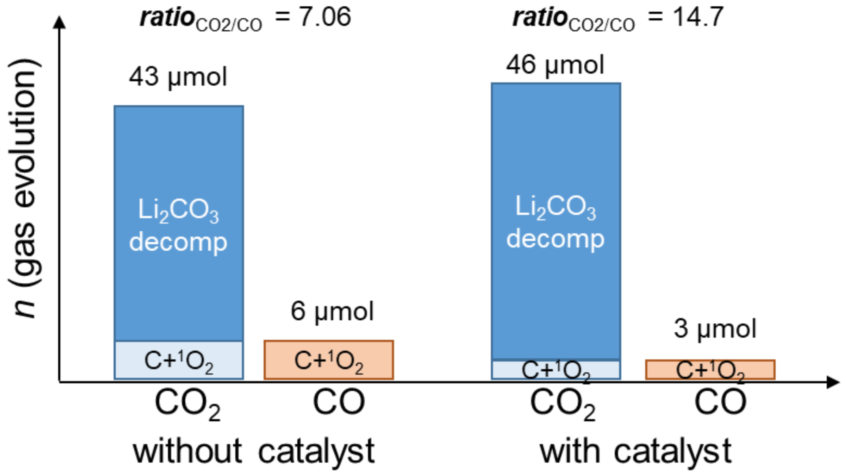

Figure S17 Bar chart of the total CO_2 and CO evolution contributed by Li_2CO_3 decomposition and carbon/electrolyte decomposition, assuming carbon/electrolyte decompose to CO and CO_2 at a ratio of 50:50.

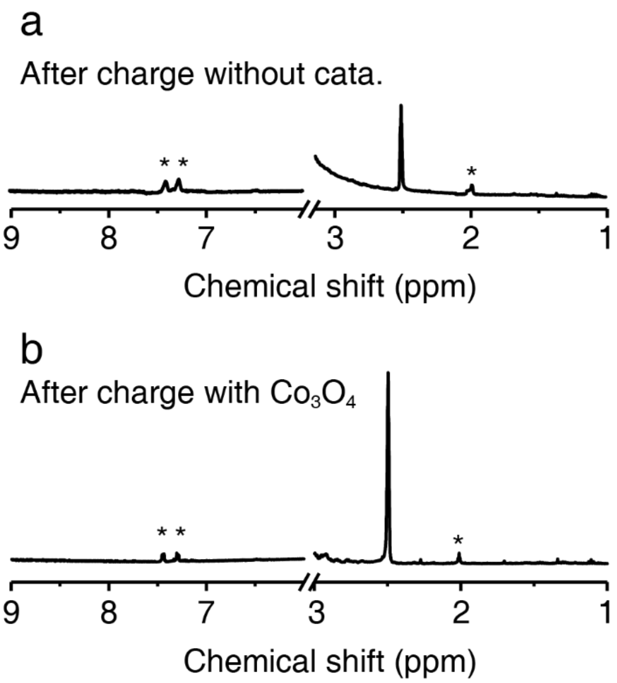

Figure S18 Comparison of singlet O_2 formation with and without a catalyst. ^{1}H NMR of the electrolyte in the cell with (a) Li_2CO_3-Super P composite electrode and (b) Li_2CO_3-Super P-Co_3O_4 composite electrode at the end of the charging process. DMA was added into the electrolyte as a molecular trap to singlet O_2. $DMAO_2$, marked with *, was identified at the end of the charging process.
